# Occurrence, Sources, and Health Risks of Chlorinated Paraffins and Environmental Persistent Free Radicals in Urban PM_2.5_ from the Megacity Shijiazhuang, China

**DOI:** 10.1021/acsomega.6c00507

**Published:** 2026-04-27

**Authors:** Shuling Duan, Wenyan Yan, Xu Yang, Fan Yang, Ke Fang, Juan Liu, Qiao Yao, Xiaoyan Dong, Na Li, Mengyao Wang, Jiajun Xiao, Xiaona Wang, Keke Long, Yibin Sun, Hongyang Cui, Qin Wang, Yi Wan, Rong Zhang, Fobang Liu, Chao Wang, Song Tang

**Affiliations:** ◧ 645576 China CDC Key Laboratory of Environment and Population Health, National Institute of Environmental Health, Chinese Center for Disease Control and Prevention, Beijing 100021, China; ‡ National Key Laboratory of Intelligent Tracking and Forecasting for Infection Diseases, 12415 Chinese Center for Disease Control and Prevention, Beijing 100021, China; § Center for Global Health, School of Public Health, Nanjing Medical University, Nanjing, Jiangsu 211166, China; ∥ Department of Environmental Science and Engineering, School of Energy and Power Engineering, 12480 Xi’an Jiaotong University, Xi’an, Shanxi 710049, China; ⊥ College of Environmental Sciences and Engineering, Beijing Forestry University, Beijing 100083, China; # School of Public Health, 12605 Shandong University, Jinan, Shangdong 250061, China; ○ School of Public Health, China Medical University, Shenyang, Liaoning 110122, China; □ College of Urban and Environmental Sciences, Peking University, Beijing 100871, China; ■ Department of Toxicology, 12553 Hebei Medical University, Shijiazhuan, Heibei 050017, China

## Abstract

Chlorinated paraffins (CPs) and environmental persistent free radicals (EPFRs) are classes of emerging organic pollutants with adverse health and environmental impacts. In this study, we investigated the occurrence, sources, environmental determinants, and health risks associated with CPs and EPFRs in PM_2.5_ collected from Shijiazhuang, China. The results demonstrated the ubiquity of both CPs (primarily SCCPs and MCCPs) and EPFRs in PM_2.5_, with concentrations ranging from 3.48 × 10^2^ to 3.89 × 10^3^ pg/m^3^ and from 1.03 × 10^12^ to 6.11 × 10^13^ spins/m^3^, respectively. Source apportionment revealed that CPs primarily originated from CP-related products, industrial emissions, and vehicular emissions, whereas EPFRs were mainly attributed to atmospheric oxidation and vehicle emissions. The concentrations of CPs and EPFRs were positively associated with PM_10_, CO, and NO_2_ and negatively associated with relative humidity. Health risk assessments indicated that children (<6 years) and adults (≥18 years) exhibited higher inhalation exposure levels to both CPs and EPFRs. Notably, individuals aged 18–59 years showed the highest cigarette equivalent inhalation exposure to EPFRs, with average values of 0.285 (head airway), 0.030 (tracheobronchial region), and 0.063 (alveolar region). These findings provide valuable scientific insights for formulating targeted control strategies and public health interventions to mitigate the health risk of these pollutants globally.

## Graphical abstract

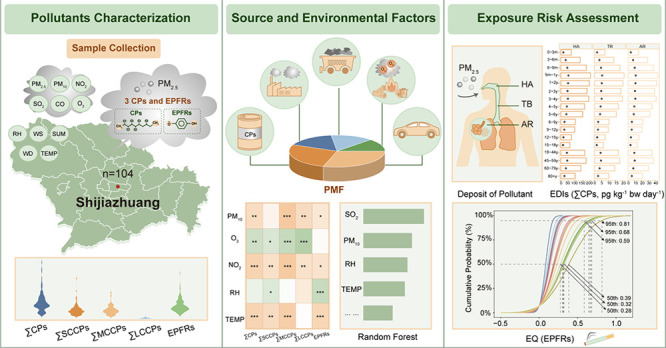

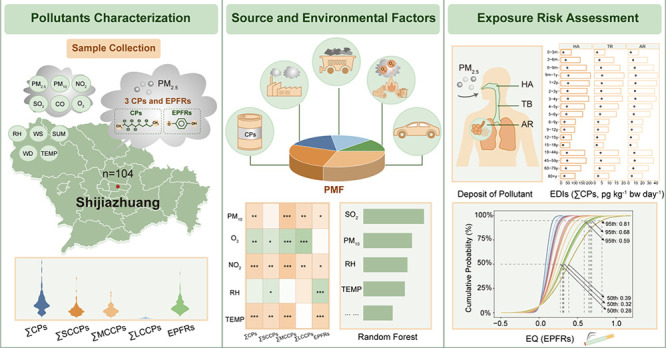

## 1 Introduction

Chlorinated paraffins (CPs) and environmental persistent free radicals (EPFRs) are recognized classes of emerging persistent organic pollutants (POPs), which have the potential to exert adverse effects on human health and environmental quality.^1,2^ CPs, classified according to carbon chain length into short-chain chlorinated paraffins (SCCPs, C_10_–C_13_), medium-chain chlorinated paraffins (MCCPs, C_14_–C_17_), and long-chain chlorinated paraffins (LCCPs, ≥C_18_),^3^ are widely used as lubricants, plasticizers, and flame retardants.^4^ Due to their potential toxicity and associated adverse effects, including endocrine disruption, asthma, and myocardial damage,^5–7^ the production and use of SCCPs and MCCPs were restricted, and they were listed under the Stockholm Convention in 2017 and 2025, respectively.^8^ EPFRs, which are stable organic free radicals found within particulate matter,^9^ are capable of continuously releasing reactive hydroxyl radicals (·OH). These ·OH-driven oxidation pathways are implicated in the degradation and environmental fate of various emerging pollutants, including CPs.^10–12^ EPFRs can induce oxidative stress upon entering the human body, potentially contributing to respiratory diseases, cardiovascular diseases, and carcinogenesis.^13–15^ However, effective control measures targeting EPFRs remain limited or lacking. Under complex environmental conditions, exposure to pollutants such as CPs and EPFRs may lead to an additive or synergistic enhancement of toxic effects, representing a notable environmental health risk.^16^

CPs and EPFRs are pervasive in various environmental matrices, including aquatic environments, land-based soils, and airborne particulate matter.^17–19^ Notably, they are frequently detected in atmospheric PM_2.5_ worldwide.^20–22^ As stated in the 2021 Global Burden of Disease report, the estimated attributable mortality from long-term exposure to ambient PM_2.5_ exceeds 4.7 million premature deaths on a global scale,^23^ with 1.9 million of these fatalities occurring in China.^24^ PM_2.5_ is characterized by small particle size, large surface area, and complex chemical composition, allowing it to function as a carrier for various adsorbed pollutants, thereby enabling deep penetration into the respiratory tract and amplifying their health impacts.^25,26^ Therefore, the accurate measurement of organic pollutant content in PM_2.5_ is crucial for assessing human health risks. Current human health risk assessments for CPs in human populations often rely on existing reference toxicity thresholds.^27^ However, there is no internationally recognized methodology for evaluating the health risks of EPFRs in PM_2.5_. Given that the electron paramagnetic resonance spectroscopy (EPR) signals and g-factors of EPFRs in PM_2.5_ are highly similar to those in cigarette tar,^2,28^ for which the health risks are well-established, we hypothesize that the inhalation of EPFRs in PM_2.5_ may pose health risks comparable to those associated with exposure to cigarette smoke. While some studies have addressed the health effects of atmospheric CPs and EPFRs, most of these efforts have primarily focused on health risk assessments in adult populations,^29–31^ often neglecting vulnerable groups such as children, infants, and the elderly. An integrated approach that encompasses the entire life cycle and addresses multiple pollutants is critical. This integrated approach would facilitate the refinement of relevant toxicological parameters, leading to improved and more accurate human health risk assessments.^32^

Atmospheric CPs primarily originate from CP-related industrial activities. Global cumulative CP emissions reached 5.2 million metric tons by 2020, with approximately 10% released into the atmosphere and China being a major contributor, accounting for 38% of the total.^4^ Environmental factors (e.g., temperature [TEMP]) influence the fate and transport of the CPs. For example, Wang et al. found that the ambient concentrations of SCCPs were positively correlated with TEMP.^33^ South et al. concluded that most MCCPs and nearly all LCCPs are transported over long distances via carriers such as particulate matter, microplastics, and nanoplastics.^34^ EPFRs primarily arise from combustion and thermal processes and can participate in atmospheric reactions facilitated by water molecules and ultraviolet radiation.^28^ Consistent with observations for CPs, Xu et al. reported a significant positive correlation between EPFRs within atmospheric PM_2.5_ and TEMP.^35^ Consequently, understanding the sources and shared environmental behaviors of CPs and EPFRs in PM_2.5_ is essential for effective pollution control and the mitigation of associated health risks.

Shijiazhuang, a northern megacity in China with a population exceeding 11 million, faces severe PM_2.5_ pollution. In 2022, the annual average concentration of PM_2.5_ in Shijiazhuang was 46 μg/m^3^,^36^ far exceeding the World Health Organization guideline value of 5 μg/m^3^.^37^ During the heating season, increased coal consumption for indoor heating not only elevates PM_2.5_ levels to approximately 1.4 times those in the nonheating season^38^ but also serves as an important source of ambient EPFRs. Furthermore, as a major industrial city in the Beijing–Tianjin–Hebei region, Shijiazhuang possesses well-developed chemical, building material, and electronic manufacturing industries, all of which extensively produce and utilize CPs-containing products.^39^ It is estimated that approximately 9.78 tons of CPs were emitted into the atmosphere from the province where Shijiazhuang is located between 2010 and 2014.^40^ Therefore, this study selected Shijiazhuang as the sampling location. The objectives of this study are (1) to characterize the distribution patterns of CPs and EPFRs in PM_2.5_ during the heating periods in Shijiazhuang; (2) to analyze the contributions of main sources to CPs and EPFRs in PM_2.5_ using source apportionment modeling; (3) to investigate the influence of atmospheric pollutants and meteorological factors on the concentrations of CPs and EPFRs; and (4) to assess the potential health implications of inhaling CPs and EPFRs across 17 age demographics, utilizing toxicological parameters.

## 2 Materials and Methods

### 2.1 Sample Collection

A total of 104 ambient PM_2.5_ samples were collected in Shijiazhuang from November 2021 to March 2022. The sampling site, representative of a typical urban air environment, was located in an area bordered by industrial zones, residential areas, and major traffic routes. Prior to sampling, quartz fiber filters were baked in a muffle furnace at 500 °C to remove residual organic compounds. PM_2.5_ samples were collected on the filters using a high-volume air sampler at a constant flow rate of 1.05 m^3^/min for 24 h. The daily meteorological parameters and concentrations of atmospheric pollutants were collected from the nearest environmental observation station, located within a 3-km radius. Specific details regarding the sampling procedures are described in our previous study.^41^

### 2.2 Pretreatment and Analysis of CPs

A modified QuEChERS (quick, easy, inexpensive, effective, rugged, and safe) method was used for sample extraction and cleanup. A quantity of quartz fiber filter, equivalent to one-eighth of the total, was meticulously divided into minute pieces and subsequently placed into an amber glass vial. A ceramic homogenizer rotor and 5 ng of internal standard (^13^C_10_-anti-Dechlorane Plus; Cambridge Isotope Laboratories, MA, USA) were sequentially added, followed by 20 mL of a mixed solvent (acetonitrile: *n*-hexane: dichloromethane, 1:1:1, v/v/v), 2 g of MgSO_4_, and 0.5 g of NaCl. The mixture was vortexed for 10 min; the procedure was continued with centrifugation at 2400× rpm to collect the supernatant. Then, 900 mg of MgSO_4_ and 150 mg of PSA were added to the supernatant, and the extraction and cleanup procedure was repeated twice. Finally, the extract (approximately 1 mL) was filtered, evaporated to dryness under a gentle stream of nitrogen, and reconstituted in 200 μL of acetonitrile before instrumental analysis. Analysis of CPs was conducted using an Ultimate 3000 Ultra-Performance Liquid Chromatography system (Thermo Fisher, CA, USA) coupled to a Q Exactive Plus Orbitrap Mass Spectrometer (Thermo Scientific, CA, USA). Analytes were separated on a Poroshell 120 EC-C_8_ column (100 × 2.1 mm, 2.7 μm). The method detection limits (MDLs) were determined by analyzing seven replicates of extracted matrix samples spiked at concentrations ranging from 2 to 5 times the estimated MDLs, with calculations based on a signal-to-noise ratio (S/N) of 3. The resulting MDLs were 0.29 ng/m^3^ for SCCPs, 0.03 ng/m^3^ for MCCPs, and 0.01 ng/m^3^ for LCCPs (Tables S1 and S2). Method accuracy and precision were evaluated through recovery experiments at three spiking levels (2, 10, and 20 ng), yielding average recoveries of 77.38%–101.39% and relative standard deviations of 2.90%–12.84% (Table S3).

### 2.3 Pretreatment and Analysis of EPFRs

The quantitative analysis of EPFRs in PM_2.5_ was described in detail in our previous work.^42^ Briefly, EPR was utilized to quantitatively analyze EPFRs in filtered samples. A total of three punches (1.2 cm^2^ per punch) from each filter were inserted into a quartz tube (5 mm inner diameter, SP Wilmad-LabGlass) and analyzed using an EPR spectrometer (A300–9.5/12, Bruker). To enhance S/N, the EPR spectra underwent baseline correction, and a fit with a Gaussian function employing the least-squares method was subsequently conducted. The concentrations of EPFRs under investigation were determined by integration of the peak area and comparison to a calibration curve. The calibration curve was generated using a standard of 4-hydroxy-2,2,6,6-tetramethylpiperidine-1-oxyl.

### 2.4 Positive Matrix Factorization Model

The positive matrix factorization (PMF 5.0) model developed by the US Environmental Protection Agency was utilized to apportion the sources of CPs and EPFRs in this study. The input data encompassed the concentrations and associated uncertainties of total CPs, SCCPs, MCCPs, LCCPs, EPFRs, SO_2_, NO_2_, CO, O_3_, PM_2.5_, organic carbon fractions (OC1, OC2, OC3, OC4), elemental carbon fractions (EC1, EC2, EC3), and the 12 elements. The calculation of the uncertainties of each variable was performed using the following equation: uncertainty = *K* × concentration. For total CPs, SCCPs, MCCPs, LCCPs, EPFRs, OC, and EC, *K* was set at 10%.^42^ For regularly monitored atmospheric pollutants and elements, *K* was set at 15%.^43^ Further details of the model setup and diagnostics are provided in Texts S5 and S6 and Tables S4–S12.

### 2.5 Risk Assessments

To evaluate the possible health hazards associated with CPs and EPFRs in atmospheric PM_2.5_, we first considered the retention of particulate matter by respiratory tract structures. We employed the international commission on the radiological protection model to explore the deposition flux (DF) of particulates in the head airway (HA), tracheobronchial region (TB), and alveolar region (AR)^44^ (as detailed in Text S4 and Table S13). For CPs, we calculated the estimated daily intake (EDI, pg kg^–1^ bw day^–1^), hazard quotient (HQ, unitless), and margin of exposure (MOE, unitless) for each type of CPs, as per eqs 1, 2, and 3, respectively.

1 $$EDI=\frac{C\times IR\times T}{BW}$$

2 $$HQ=\frac{EDI}{TDI}$$

3 $$MOE=\frac{NOAEL}{EDI}$$

The concentration of CPs in PM_2.5_ is denoted as C (pg/m^3^). The inhalation rate (IR, m^3^/day) and body weight (BW, kg) for adults and children were ascertained from the Chinese Exposure Factors Handbook^45,46^ (Table S14). The average daily exposure time (T) denotes the outdoor exposure time. The tolerable daily intake (TDI) for noncarcinogenic effects of SCCPs, MCCPs, and LCCPs was set at 100 μg kg^–1^ day^–1^.^47^ The no observed adverse effect levels (NOAELs) for SCCPs, MCCPs, and LCCPs were 10, 23, and 100 mg kg^–1^ day^–1^,^48^ respectively. For EPFRs, the daily exposure dose (DED, spins kg^–1^ bw day^–1^) and the number of equivalent cigarettes (EQ, unitless) related to daily EPFRs exposure were calculated using eqs 4 and 5:

4 $$DED=\frac{{RC}_{PM}\times IR\times EF}{BW}$$

5 $$EQ=\frac{{RC}_{PM}\times IR}{{RC}_{cig}\times C_{tar}}$$

RC_PM_ represents the concentration of EPFRs in PM_2.5_ (spins/m^3^); EF denotes the outdoor exposure fraction (unitless), where EF is equivalent to T. RC_cig_ indicates the concentration of free radicals in cigarette tar (4.75 × 10^16^ spins/g tar). C_tar_ refers to the tar content per cigarette (0.013 g tar/cigarette).^29^ When considering the retention of particulate matter in the respiratory tract, DF × EDI, DF × DED, and DF × EQ were utilized in the calculations.

### 2.6 Data Analysis

All statistical analyses were performed using R software (version 4.3.1). The Kruskal–Wallis H test was performed to evaluate concentration differences among types of CPs (SCCPs, MCCPs, and LCCPs), as well as the temporal dynamics of both CPs and EPFRs concentrations. The Spearman rank correlation method, a linear mixed-effects model, and a random forest model were employed to examine correlations among pollutants, analyze the relationships between environmental factors and concentrations of CPs and EPFRs, and identify key environmental influences. *p*-values were adjusted for multiple comparisons via the Benjamini–Hochberg procedure with statistical significance defined by a false discovery rate (FDR) of less than 0.05. To reduce interindividual exposure uncertainties and to assess population-wide exposure risk, Monte Carlo simulations were conducted using R with 10,000 iterations based on concentrations of CPs and EPFRs in PM_2.5_. Normality was concurrently determined by the Shapiro-Wilk test (*p* > 0.05). The following R packages were utilized: reshape2, tidyverse, ggpubr, ggplot2, psych, lme4, lmerTest, sjstats, splines, car, randomForest, rfPermute, and randomForestSRC.

## 3 Results and Discussion

### 3.1 Characteristics of CPs and EPFRs in PM_2.5_

#### 3.1.1 Distribution of CPs in PM_2.5_

The detection frequencies of SCCPs, MCCPs, and LCCPs reached 100%, indicating their ubiquitous environmental distribution in Shijiazhuang. Concentrations of total CPs in PM_2.5_ ranged from 3.48 × 10^2^ to 3.89 × 10^3^ pg/m^3^, with a mean of 1.17 × 10^3^ pg/m^3^. The mean concentrations of SCCPs and MCCPs were 5.99 × 10^2^ pg/m^3^ (range: 1.60 × 10^2^–2.76 × 10^3^ pg/m^3^) and 5.31 × 10^2^ pg/m^3^ (range: 92.1–1.74 × 10^3^ pg/m^3^), respectively (Figure 1A and Table S15). Compared to cold-season measurements, the levels in this study were lower than those from Chinese cities including Beijing (SCCPs: 5.60 × 10^4^ pg/m^3^, MCCPs: 8.50 × 10^4^ pg/m^3^), Dalian (SCCPs: 5.10 × 10^3^ pg/m^3^, MCCPs: 6.70 × 10^3^ pg/m^3^), the Pearl River Delta (SCCPs: 7.57 × 10^3^ pg/m^3^, MCCPs: 7.11 × 10^3^ pg/m^3^),^27,49,50^ as well as from other countries, including Canada (SCCPs: 2.56 × 10^4^ pg/m^3^), India (SCCPs: 1.02 × 10^4^ pg/m^3^, MCCPs: 3.62 × 10^4^ pg/m^3^), and Pakistan (SCCPs: 5.13 × 10^3^ pg/m^3^, MCCPs: 4.21 × 10^3^ pg/m^3^).^51,52^ In contrast, they were higher than the values reported for Norway (SCCPs: 88.7 pg/m^3^) and Australia (SCCPs: 180 pg/m^3^, MCCPs: 160 pg/m^3^)^49,53^ (Table S16). These variations likely reflect differences in the production, usage, and distribution of CPs, as well as variations in regulatory stringency across countries and regions.

**Figure 1:**
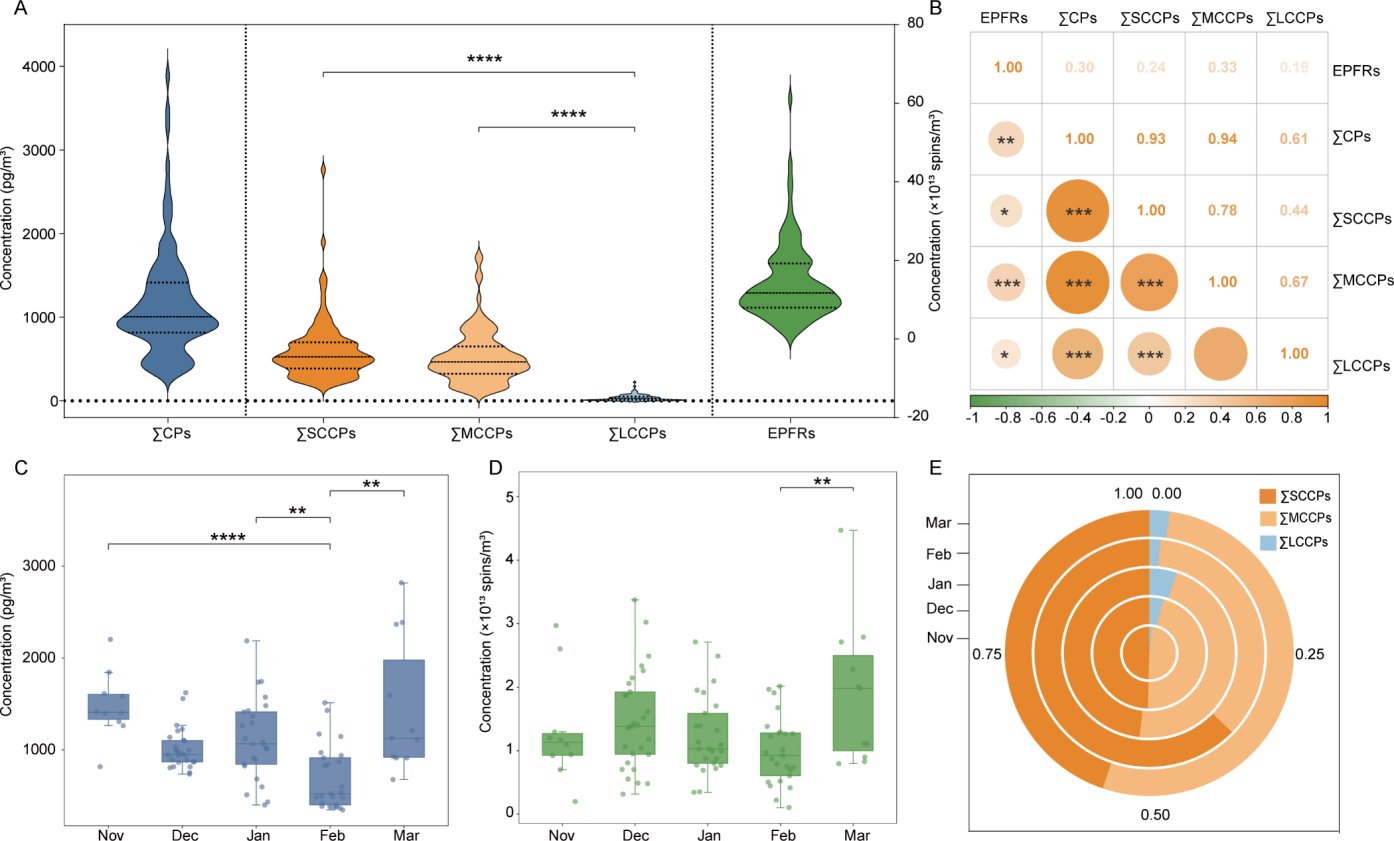
Characteristics of CPs and EPFRs in urban PM_2.5_. Violin plots depicting the concentrations of CPs and EPFRs in PM_2.5_ from Shijiazhuang (A). Correlation analysis illustrating the relationship among pollutants (B). Box plots showing temporal trends of total CPs (C) and EPFRs (D) concentrations across sampling months. Pie chart displaying the compositional ratios of SCCPs, MCCPs, and LCCPs in total CPs (E). Asterisks denote statistical significance: *p < 0.05, **p < 0.01, and ***p < 0.001.

In this study, significantly higher concentrations of SCCPs and MCCPs were found in comparison to LCCPs (*p* < 0.001). SCCPs and MCCPs were the dominant components of atmospheric CPs, accounting for approximately 51.28% and 45.47% of the total CPs burden, respectively. In contrast, LCCPs contributed only about 3.24%, likely attributable to their inherently lower volatility (Figure 1E). The mean concentrations of LCCPs were 37.9 pg/m^3^ (range: 2.05–2.22 × 10^2^ pg/m^3^) (Figure 1A and Table S15). This level is lower than the cold-season concentrations reported for the Pearl River Delta (1.95 × 10^3^ pg/m^3^), Guangzhou (1.63 × 10^3^ pg/m^3^),^27,54^ as well as those reported for Vietnam (42 pg/m^3^) and Australia (97 pg/m^3^).^49^ This finding may imply comparatively limited LCCPs sources within the investigated area. Despite fewer atmospheric reports compared to SCCPs and MCCPs, LCCPs demonstrate more pronounced metabolic disturbance effects^34^ and, due to bioaccumulation, potentially higher risks for organisms at the top of the food chain. Consequently, their continuous long-term monitoring is warranted.^55^ However, the accurate quantification of more LCCPs currently remains challenging, particularly due to the lack of internal standards that cover their entire carbon chain length distribution. Coupled with the vast number of congeners, this makes effective differentiation and quantification difficult.^56^ Therefore, the development of highly sensitive and broadly applicable analytical methods for the detection and quantitation of LCCPs is imperative for future research.

The profiles of congener group abundance of SCCPs, MCCPs, and LCCPs in PM_2.5_ are presented in Figure S1. The dominant alkane chain-length group in SCCPs was C_13_ (mean 40.96%), followed by C_11_ (mean 25.41%) and C_12_ (mean 22.89%). For MCCPs, C_14_ (mean 59.93%) was the most abundant alkane-chain length group, followed by C_15_ (mean 26.59%). In LCCPs, C_18_ (mean 41.33%) and C_19_ (mean 31.45%) were the dominant alkane chain-length groups. This carbon chain distribution is consistent with findings from the Pearl River Delta region^27^ but contrasts with reports from Pakistan,^57^ Vietnam,^49^ and Greece,^58^ where C_10–11_ congeners predominated, and from the UK, where C_12_ was more abundant.^59^ These regional disparities likely reflect differences in the commercial CP mixtures used locally. In general, both MCCPs and LCCPs exhibit a decreasing trend in carbon content with increasing alkane chain length, likely due to lower volatility associated with longer alkane chains.^34^ In terms of the chlorine pattern, Cl_6_ and Cl_7_ were the predominant chlorine constituents. A statistically significant difference (*p* < 0.0001) in chlorination degree was observed among CPs in PM_2.5_, with SCCPs exhibiting the highest levels (average 58.40%), followed by MCCPs (mean 53.68%) and LCCPs (mean 51.23%). This similarity in composition to Chinese commercial CP-42 and CP-52 mixtures^58^ indicates that the CPs found in Shijiazhuang are largely attributable to the extensive application of these two products.

#### 3.1.2 Distribution of EPFRs in PM_2.5_

In our previous research, we found that EPFRs were primarily distributed in atmospheric PM_2.5_.^42^ Therefore, we focused on detecting EPFRs in urban PM_2.5_ collected from the University of Shijiazhuang. The detection frequency of EPFRs was 100%. The mean concentration of EPFRs in PM_2.5_ was 1.43 × 10^13^ spins/m^3^ (range: 1.03 × 10^12^ to 6.11 × 10^13^ spins/m^3^, Figure 1A). This is lower than the EPFR levels observed in atmospheric PM_2.5_ during the cold seasons in Zhengzhou (2.72 × 10^15^ spins/m^3^), Dalian (2.41 × 10^15^ spins/m^3^), Xi’an (3.17 × 10^14^ spins/m^3^), Yuncheng (2.82 × 10^15^ spins/m^3^), Mongolia (8.87 × 10^13^ spins/m^3^, and Pakistan (1.20 × 10^14^ spins/m^3^).^20,60–64^ However, the EPFR concentration measured in Beijing (1.42 × 10^13^ spins/m^3^)^20^ was found to be comparable to our findings. This observed similarity warrants further investigation into potential regional influences such as atmospheric transport patterns and emission profiles (Table S17). Overall, the EPFR concentrations at this sampling site were lower than in most other urban areas, suggesting that the sources contributing to EPFRs at the study area are relatively limited compared to other cities. Nevertheless, in comparison with rural areas in Quzhou (4.49 × 10^12^ spins/m^3^),^42^ the mean EPFR concentrations in this study were found to be an order of magnitude higher. The elevated EPFR levels in urban areas are likely attributable to more intensive anthropogenic sources, such as denser industrial activities and vehicular emissions. The g-factor is a key parameter for characterizing EPFRs. A g-factor of less than 2.0030 is indicative of carbon-centered radicals (e.g., aromatic hydrocarbon radicals),^65^ whereas a g-factor of greater than 2.0040 corresponds to oxygen-centered radicals (e.g., semiquinone radicals).^66^ The g-factors of PM_2.5_ samples exhibited a range from 2.0033 to 2.0040, with mean value of 2.0036 ± 0.0001, indicating the coexistence of carbon-centered and oxygen-centered radicals or the presence of heteroatoms adjacent to the carbon-centered radicals.^20^

Although the absolute concentration levels of CPs and EPFRs cannot be directly compared due to their different units, a significant positive correlation was observed between them (Figure 1B), suggesting potential common sources and warranting further investigation. When comparing CP and EPFR concentrations with those reported in other cities, methodological differences may introduce systematic bias. For CPs, different ion sources show distinct responses toward CP homologues. Moreover, the lack of commercial isotopic standards leads to the use of various surrogate internal standards (e.g., ^13^C_10_-anti-Dechlorane Plus, ^13^C_10_-trans-chlordane, and ^13^C_10_-BDE 209), introducing quantitative uncertainty. To improve data comparability, we advocate unified international analytical standards for CPs to facilitate reliable assessment of their global pollution levels and health risks.

### 3.2 Source and Relationship of CPs and EPFRs in PM_2.5_

To further investigate the sources of pollutants and quantify their contributions, PMF analysis was performed (Figure 2A). In conjunction with our previous studies^42^ and the correlation of atmospheric pollutants with related industries, we identified five major source factors (Text S6 and Tables S4–S12.). Factor 1, exhibiting substantial contributions from OC3, OC4, EC1, EC2, EC3, Mn, Cu, and NO_2_, is primarily attributed to vehicle emissions. Specifically, OC3 and OC4 primarily originate from gasoline vehicle emissions, whereas EC1, EC2, and EC3 are distinctly associated with diesel vehicle emissions.^67^ Mn is attributable to gasoline additives, and Cu originates from brake wear.^68^ NO_2_ is commonly associated with vehicle emissions.^69^ Factor 2, characterized by a high proportion of CPs, is attributed to their emission during the production and use of related products.^3^ Factor 3 reflects contributions from burning processes and atmospheric oxidation processes, as evidenced by the significant proportion of OC, EC, and SO_2_. For example, OC1 and OC2 are identified as tracers for biomass burning, and SO_2_ is significantly emitted from various waste incineration processes.^70^ Furthermore, this factor shows a significant presence of O_3_, reflecting atmospheric oxidation processes and capacity.^71^ Factor 4 is associated with coal combustion, with As, Pb, CO, and NO_2_ serving as typical source tracers.^72,73^ Factor 5 is attributed to industrial emissions, characterized by substantial loadings of metallic elements including Fe, Al, Cr, and Ni. These elements are indicative of iron and steel production and nonferrous metal smelting activities.^74^

**Figure 2:**
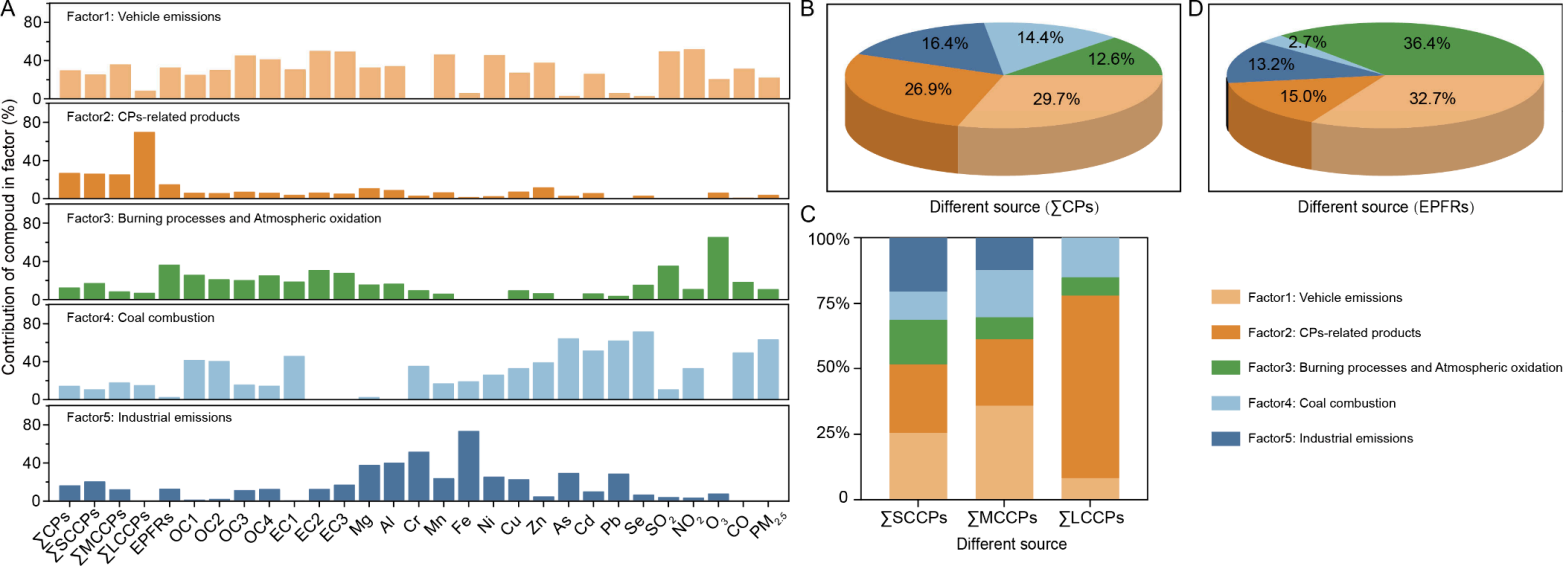
Source of CPs and EPFRs in PM_2.5_. Bar graphs showing factor profiles of CPs and EPFRs in PM_2.5_ identified by PMF analysis; colored bars indicate key indicator compounds for each factor (A). Pie and stacked bar charts showing the relative contributions of three factors to CPs and EPFRs (B–D).

#### 3.2.1 Source of CPs in PM_2.5_

Factor 1, associated with vehicle emissions, contributed 29.7%, 25.5%, 35.8%, and 8.2% to the source apportionment for CPs, SCCPs, MCCPs, and LCCPs, respectively (Figure 2B), suggesting that vehicle-related industries are also major contributors to atmospheric CPs. Brahney et al. demonstrated that the mechanical breakdown of tire and brake rubber produces small particles (tire wear particles, TWPs) capable of mobilizing CPs and reintroducing them into the atmosphere via roadside energy.^75^ Extending this, Du et al. evaluated tire mass and simulated air emissions, revealing that TWP emissions constitute 20% of total CP emissions during a tire’s lifecycle, compared to 6% from direct air emissions. This underscores TWPs as the predominant pathway for CP dispersal, exhibiting significantly higher transport efficiency relative to other CP-containing products.^76^ Factor 2 and Factor 5, primarily associated with CP-related products and industrial emissions, collectively accounted for 43.3% (Factor 2:26.9%, Factor 5:16.4%), 46.7% (Factor 2:26.1%, Factor 5:20.6%), 37.8% (Factor 2:25.4%, Factor 5:12.3%), and 69.7% (solely from Factor 2) of the source apportionment for total CPs, SCCPs, MCCPs, and LCCPs, respectively (Figure 2B,C). This indicates that industrial emissions are likely the dominant source of atmospheric CPs. Given the absence of known natural sources of CPs, their atmospheric presence is primarily attributed to anthropogenic releases related to the production, storage, transport, manufacturing, and application of CP-containing products.^3,77^ Metalworking fluid mixing/formulation is a significant source of CP release during production, with estimates suggesting a loss of 1–2% of the total CPs used to the environment.^78^ Furthermore, releases during polymer processing are estimated at 0.1%, and volatilization rates of SCCPs and MCCPs from rubber products during their service life are around 0.05% (United Nations Environment Programme).^79^ Despite the relatively low contributions of Factor 3 (burning processes, 12.6%) and Factor 4 (coal combustion, 14.4%) (Figure 2B), high concentrations of CPs were detected in both fly ash and bottom ash produced during the landfilling and incineration of e-waste.^80^ The presence of large e-waste processing facilities in the southeastern part of the study area, coupled with the identified characteristic of southeastern wind recirculation in back-trajectory analyses,^41^ suggests that pollution sources in this direction may influence CP contamination within the region. In addition, although coal combustion is not a direct emission source of CPs, it contributes significantly to local PM_2.5_ levels, accounting for up to 63% (Figure 2A). This suggests that coal combustion may indirectly facilitate the partitioning and transport of CPs in the atmosphere by contributing to the PM_2.5_ formation. PM_2.5_ acts as a crucial carrier for CPs, scavenging these compounds during formation and transport, which explains the indirect link between coal combustion and ambient CP levels.

#### 3.2.2 Source of EPFRs in PM_2.5_

EPFRs in air originate from sources more extensive than CPs. Various combustion processes have the potential to emit EPFRs into the atmosphere.^28^ In this study, Factor 3, attributed to burning processes and atmospheric oxidation, constituted the primary source of EPFRs in PM_2.5_, contributing 36.4% to their overall source apportionment (Figure 2D). In rural areas, the combustion of biomass, such as firewood, is a common heating method. The emissions from this process can be transported by airflow to urban areas, potentially impacting urban air quality, although this influence is generally relatively minor. Atmospheric oxidation plays a crucial role in transforming EPFRs. Mechanistically, EPFRs are known to be generated through photochemical reactions involving ozone and particulate matter in the air via two principal pathways: first, solar radiation and O_3_ can directly cleave stable covalent bonds within organic matter, leading to EPFRs formation;^81^ second, metals can act as electron acceptors, facilitating electron transfer from organic compounds such as polycyclic aromatic hydrocarbons, thereby forming EPFRs.^82^

Subsequently, Factor 1, Factor 5, and Factor 4 were attributed to vehicle emissions, industrial emissions, and coal combustion, contributing 32.7%, 13.2%, and 2.7%, respectively (Figure 2D). According to Zhao et al., multiple combustion sources, such as wood, diesel, coal, and solid waste burning, contribute a large fraction of EPFRs found in PM_2.5_; moreover, their study revealed that vehicle emissions are a significant source of EPFRs in PM_2.5_ present in the air.^2^ Oyana et al. analyzed 107 leaf samples and reported that the 10 highest EPFRs concentrations (up to 3.68 × 10^19^ spins/g) correlated with areas primarily impacted by small vehicles, diesel trucks, and major industrial locations. These findings suggest a strong link between vehicle emissions and EPFRs levels in PM_2.5_.^83^ Our sampling site is located in the city center, characterized by dense commercial and residential activities and the proximity to several major arterial roads. This explains why vehicular emissions represent a significant source of EPFRs. In addition, the processes of coalification,^84,85^ mining,^86^ and transportation^87^ can potentially release EPFRs into the atmospheric environment. Coal combustion has become accepted as an important contributor to atmospheric EPFRs levels.^81,88^ However, the contribution of coal combustion to EPFRs in our study area was relatively minor. This observation is likely attributable to the widespread implementation of clean heating initiatives in recent years, such as the replacement of coal with natural gas (gas-for-coal) and electricity (electricity-for-coal) for heating purposes in the region. Factor 2, attributed to CP-related products, contributed 15% to the sources of EPFRs (Figure 2D). This contribution is likely linked to the end-of-life disposal phase of CP-related products, particularly thermal treatment and incomplete combustion, which are known to generate EPFRs.^9^

Additionally, given the inherent oxidative properties of EPFRs, there might be other correlations between the CPs and EPFRs. For example, EPFRs may influence the atmospheric degradation of CPs. Photodegradation, which is mediated by highly oxidative hydroxyl radicals (·OH) is a key pathway for CPs degradation.^89,90^ Notably, EPFRs attached to PM_2.5_ can react with oxygen and undergo photolysis under sunlight irradiation, leading to generation of ·OH.^21^ These ·OH radicals could participate in the degradation of CPs. If this hypothesis holds true, such interactions suggest potential antagonistic effects between EPFRs and CPs in the formation and persistence of air pollutants. However, Spearman correlation analysis revealed a significant positive correlation between CPs and EPFRs (*r*_CPs_ = 0.30, *r*_SCCPs_ = 0.24, *r*_MCCPs_ = 0.33, *r*_LCCPs_ = 0.19, all *p* < 0.05; Figure 1B). This finding contradicts the initial hypothesis. Further quantitative analysis demonstrated that EPFRs-mediated ·OH production contributes less than 0.03% to the steady-state atmospheric ·OH concentration, which is insufficient to drive significant CP degradation (Text S7). PMF model results indicated that CPs and EPFRs share common significant emission sources, such as vehicle emissions and industrial discharges. These similar “source effects” likely dominated their atmospheric concentration distributions, thereby masking the potential negative correlation signal that would arise from EPFR-mediated oxidative degradation of CPs. Moreover, compared with SCCPs and LCCPs, MCCPs exhibited the strongest correlation with EPFRs (*r* = 0.33, *p* < 0.001), suggesting that MCCPs may be more suitable as indicators for tracing EPFRs emitted from industrial combustion processes. Moving forward, given the complexity of combustion-derived sources of CPs and EPFRs in the study area, future research should conduct year-round continuous observations combined with high-time-resolution source apportionment techniques to precisely identify their emission sources and environmental fate processes.

### 3.3 CPs and EPFRs: Associations with Environmental Drivers

Atmospheric distribution and concentration levels of pollutants are influenced by meteorological conditions and the presence of other atmospheric pollutants through their effects on phase partitioning, dispersion, and accumulation.^91^ In this study, we investigated the concentrations of CPs and EPFRs in the atmosphere and analyzed their correlations with both regularly monitored atmospheric pollutants (e.g., PM_2.5_, PM_10_, CO, O_3_, NO_2_, and SO_2_) and meteorological factors (e.g., solar radiation [SUN], wind direction [WD], wind speed [WS], relative humidity [RH], and TEMP). Furthermore, to evaluate the key environmental determinants affecting CPs and EPFRs in PM_2.5_, environmental drivers were categorized into 12 principal components by using a random forest model.

#### 3.3.1 Relationships of CPs and EPFRs in PM_2.5_ with Atmospheric Pollutants

The present study demonstrated a predominantly positive correlation between the concentrations of CPs and EPFRs and regularly monitored atmospheric pollutants, as shown in Figure 3A. The concentrations of CPs and EPFRs exhibited significant positive correlations with particulate matter concentrations (*r*_s_ = 0.3 and 0.2, respectively, *p* < 0.05, Figure 3A,B, Figures S2–S4), suggesting that atmospheric particulate matter could potentially facilitate the long-range transport of these pollutants. Owing to their small particle size and large specific surface area, PM_10_ and PM_2.5_ can effectively adsorb semivolatile organic compounds and POPs with low vapor pressure and high molecular polarity.^92^ This adsorption capability influences the environmental transport, transformation, and ultimate fate of these pollutants. Therefore, strengthening the regulation of atmospheric particulate matter should be considered a viable strategy for the co-control of CP and EPFR pollution. Additionally, the concentrations of CPs and EPFRs were positively correlated with CO, NO_2_, and SO_2_, with the strongest correlation observed for NO_2_. Specifically, correlation coefficients (*r*) for SCCPs, MCCPs, LCCPs, and EPFRs with NO_2_ were 0.27, 0.47, 0.26, and 0.17, respectively (*p* < 0.05) (Figure 3A,B), implying a potential common source for these pollutants and NO_2_, such as vehicular emissions.^93^ Consequently, targeted management of traffic emissions, such as through the widespread integration of new energy vehicles, may reduce ambient concentrations of CPs and EPFRs by limiting their primary sources. Notably, CPs showed a significant negative correlation with O_3_ (*r*_s_ = −0.32, *p* < 0.05) (Figure 3A, Figures S2–S4). This inverse relationship is likely explained by the removal of CPs via O_3_-dominated atmospheric oxidation. O_3_, a potent oxidant, can photolytically produce ·OH radicals, which then initiate chain oxidation and degradation of CPs,^34,94^ effectively destroying their chemical structure and lowering environmental concentrations.

**Figure 3:**
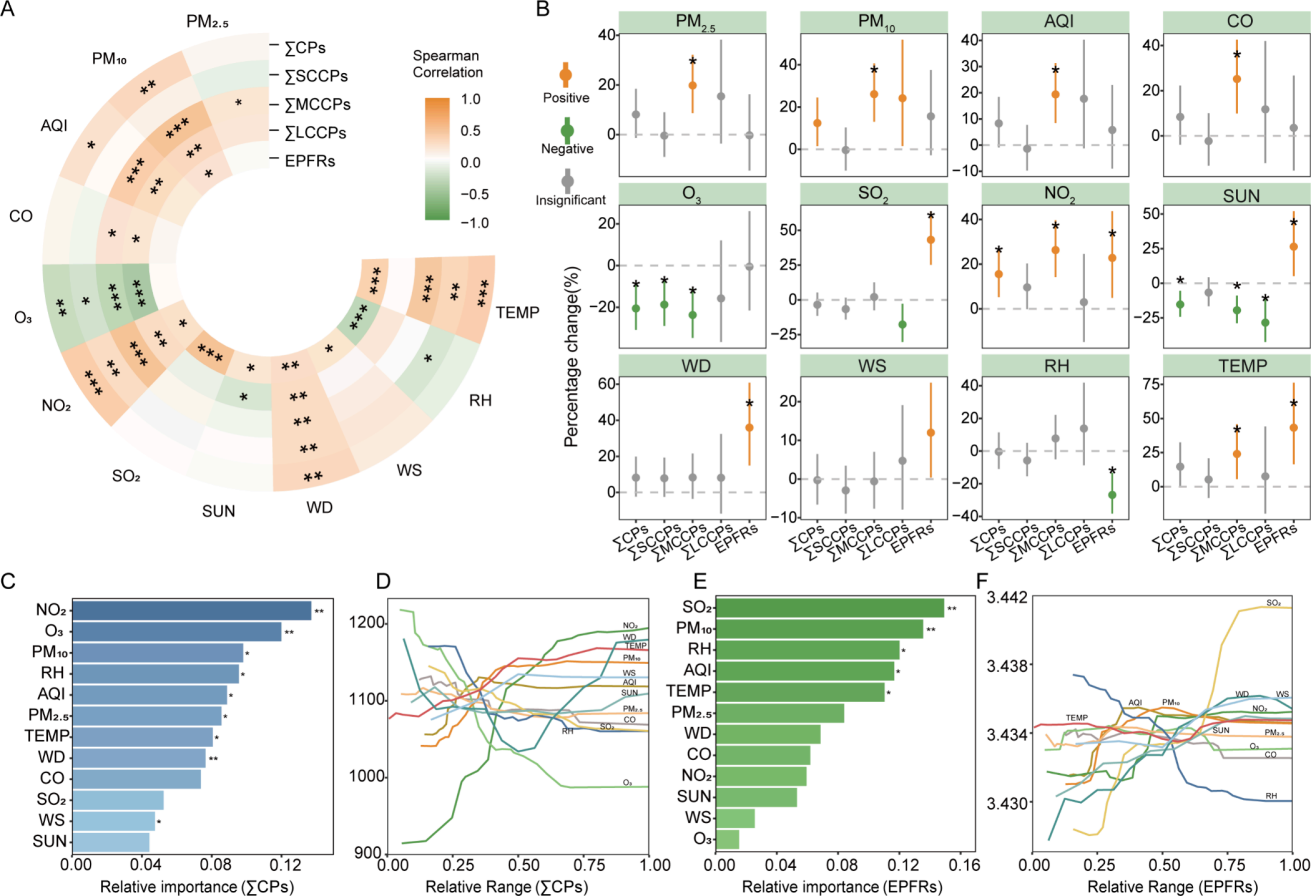
Environmental drivers of CPs and EPFRs in PM_2.5_. Spearman’s correlation heatmap showing the relationships between environmental factors and concentrations of CPs and EPFRs (A). A linear mixed effects model demonstrating the association of specific environmental factors with concentrations of CPs and EPFRs (B). Bar graphs of the relative importance (C and E) and marginal-effect line plots across the 0–100th percentile range (D and F) for environmental drivers of CPs and EPFRs in PM_2.5_, derived from the random forest analysis. Asterisks denote statistical significance: *p < 0.05, **p < 0.01, and ***p < 0.001. Abbreviations: TEMP, temperature; RH, relative humidity; WS, wind speed; WD, wind direction; SUN, sunshine duration.

#### 3.3.2 Relationships of CPs and EPFRs in PM_2.5_ with Meteorological Factors

Meteorological factors also influence the atmospheric concentrations of CPs and EPFRs. SUN was directly proportional to EPFRs (*r*_s_ = 0.19, *p* < 0.05; Figure 3A), potentially due to the role of sunlight in EPFR generation. Specifically, under light irradiation, transition metals can catalyze the production of reactive oxygen species, which subsequently promotes the conversion of radical cations to oxygenated EPFRs.^95^ WS and WD also impact atmospheric pollutant transport, influencing their dispersion and dilution. Atmospheric CPs and EPFRs exhibited positive correlations with both WD and WS (Figure 3A,B). In the study area during the winter and spring, prevailing northwesterly winds transported pollutants from upwind western industrial zones. Elevated wind speeds may further enhance this transport, leading to increased concentrations of CPs and EPFRs in the study area. Frequent dust events occur in the sampling area during the winter and spring. TEMP exhibited a moderate positive correlation with atmospheric concentrations of CPs and EPFRs (Figure 3A), a phenomenon that can be attributed to constrained gas-particle partitioning at low temperatures.^33^ Instead, rising temperatures primarily promote CP volatilization from surface particles and wind-driven dust resuspension, thereby entraining contaminant-laden particles into the atmosphere.^96^ Conversely, a substantial negative correlation was identified between atmospheric CP and EPFR concentrations and RH (Figure 3A). This could be attributed to the increased water content in the air associated with higher RH, leading to hygroscopic growth of particles.^97^ The resulting increase in particle weight promotes deposition and reduces atmospheric pollutant concentrations. Consistent with this, Jia et al. observed a sharp decline in EPFR formation rates above 11% RH, indicating that elevated water content inhibits EPFRs formation.^98,99^ Furthermore, Nwosu et al. reported a significantly enhanced EPFR decay rate at higher RH, with a 5-fold increase at 75% RH compared to 22–38% RH, and a 71-fold increase compared to vacuum.^100^

To further clarify the relative importance of environmental factors, we used a random forest model to measure the impact that atmospheric pollutants and meteorological factors had on CPs and EPFRs. The analysis revealed that CP concentrations were primarily governed by NO_2_, O_3_, PM_10_, RH, AQI, and PM_2.5_, indicating their strong association with CPs levels (Figure 3C,D). However, EPFR levels were influenced by SO_2_, PM_10_, RH, AQI, and TEMP (Figure 3E,F). Although correlation analyses using meteorological factors from the winter and spring seasons have certain limitations, identifying potential meteorological influences during periods of elevated pollutant concentrations can provide a basis for pollution control during specific periods. Future year-round monitoring is necessary to further elucidate the complex interactions among air quality, meteorological conditions, and persistent pollutants. A comprehensive understanding of these relationships is crucial for formulating effective pollution reduction strategies and optimizing targeted interventions aimed at safeguarding human health.

### 3.4 Health Risks of CPs and EPFRs in PM_2.5_ through Inhalation Exposure

Atmospheric PM_2.5_ plays a crucial role in human exposure to organic pollutants via inhalation, facilitating the transport of these pollutants deep into the respiratory system and posing risks to human health. The specific equations and parameters are presented in Text S3 and Tables S18–S20. To quantify the potential health risks from these PM_2.5_-bound pollutants, we calculated the EDIs, HQs, and MOEs for CPs in atmospheric PM_2.5_,^27^ as well as the DEDs and EQs of EPFRs from inhaled atmospheric PM_2.5_.^29^ Calculations were performed for 17 age groups, specifically: 0–3 months, 3–6 months, 6–9 months, 9 months–1 year, 1–2 years, 2–3 years, 3–4 years, 4–5 years, 5–6 years, 6–9 years, 9–12 years, 12–15 years, 15–18 years, 18–44 years, 45–59 years, 60–79 years, and over 80 years, based on the definitions provided in the *Chinese Exposure Factors Handbook*.^43^

#### 3.4.1 Health risks of CPs in PM_2.5_ through Inhalation Exposure

We determined the EDIs, HQs, and MOEs to CPs in atmospheric PM_2.5_ by age groups (Table S18). The resulting EDIs ranged from 17.74 to 57.57 pg kg^–1^ bw day^–1^ for total CPs, with values of 9.29–30.13 pg kg^–1^ bw day^–1^ for SCCPs, 8.22–26.69 pg kg^–1^ bw day^–1^ for MCCPs, and 0.5–1.64 pg kg^–1^ bw day^–1^ for LCCPs. These EDIs were substantially lower than the TDI of 100 μg kg^–1^ day^–1^ established by the International Programme on Chemical Safety,^47^ suggesting a considerable margin of safety. A comparison of our EDI estimates with previous studies revealed that EDIs in Shijiazhuang were lower than those reported in Guangzhou (8.71 × 10^4^ to 2.38 × 10^5^ pg kg^–1^ bw day^–1^)^50^ and the Pearl River Delta (230 to 1.84 × 10^4^ pg kg^–1^ bw day^–1^).^27^ These discrepancies are likely attributable to variations in the calculation parameters and regional CPs concentrations. By incorporating the proportion of outdoor activity for the Chinese population over a full day (approximately 10% of the day spent outdoors), our study yielded lower EDIs compared to those calculated from studies assuming 12 h of outdoor exposure.^27^ Notably, EDIs of CPs in PM_2.5_ exhibited elevated exposure levels in both children (<6 years) and adults (≥18 years). Given that childhood represents a critical developmental window characterized by heightened susceptibility to environmental pollutants,^101^ a thorough investigation into the associated health risks in this vulnerable subpopulation is warranted. Furthermore, the considerable exposure observed in the adult demographic demands equal consideration, underscoring the necessity of implementing targeted personal protective measures during episodes of severe atmospheric pollution. Analysis of deposition patterns within the respiratory system indicated that HA region was the dominant site of CPs deposition (Figure 4A, Table S13), which is consistent with previous research by Li et al.^102^ HQs for CPs were less than 1 across all age groups (Figure 4B), suggesting that inhalation of CPs in PM_2.5_ does not pose a significant noncarcinogenic health risk to the general population in the study area. Furthermore, the MOEs calculated for all CPs exceeded 1000 (Table S20). The U.S. EPA defines MOEs below 1000 as indicative of high risk. Collectively, these findings suggest a low probability of significant carcinogenic risk to the population in the study area from CP exposure.

**Figure 4:**
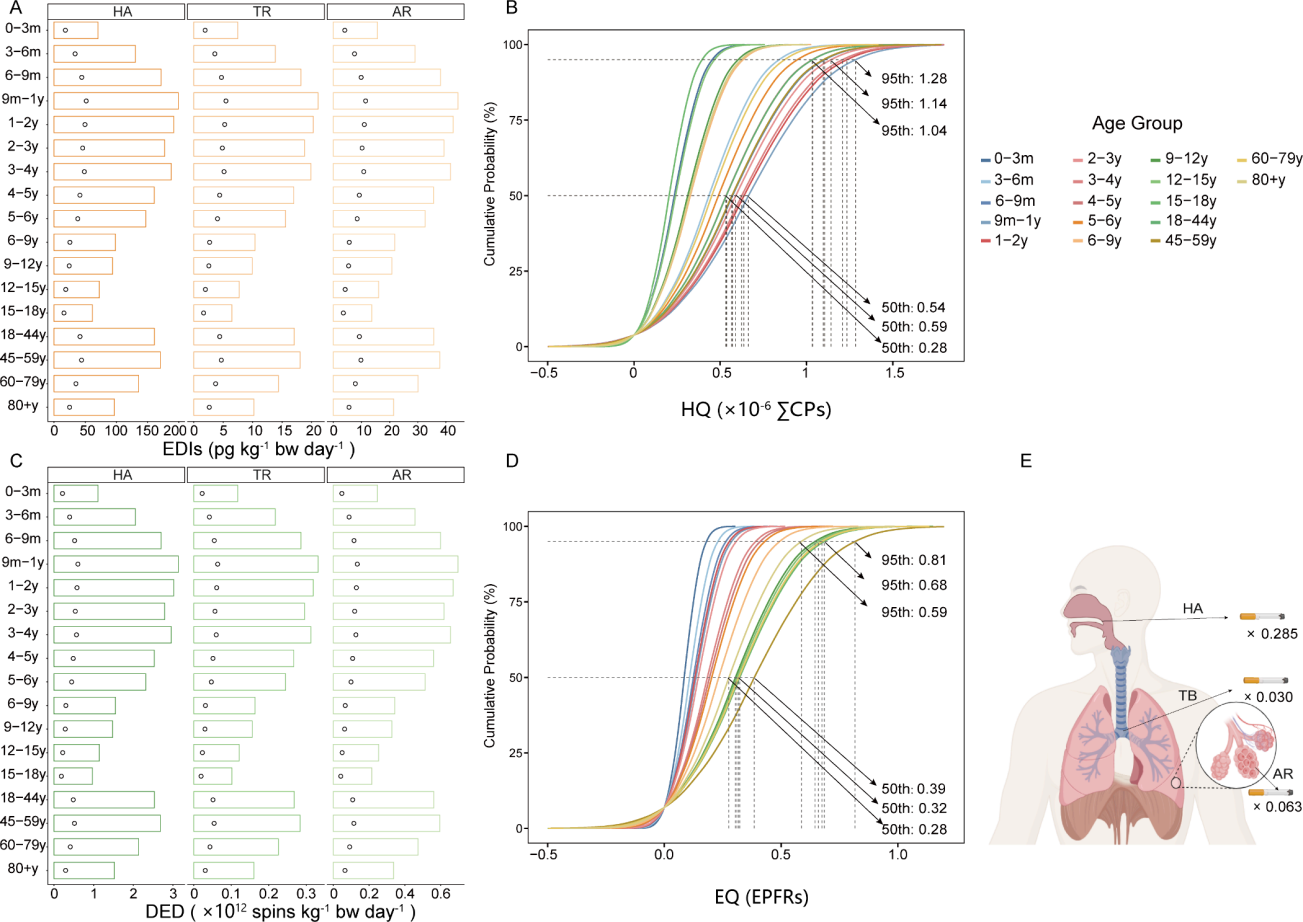
Health risks associated with CPs and EPFRs in PM_2.5_. Bar graphs showing the EDI of CPs (A) and DED of EPFRs (C) deposited in different regions of the respiratory tract across different age groups. Circles within the bars indicate the median values, and the tops of the bars represent the maximum values. Probability density distributions depict the HQ for total CPs (B) and EQ for EPFRs (D) among the different age groups. PAnel E presents the estimated number of cigarette equivalents corresponding to daily inhalation of EPFRs in PM_2.5_ across various respiratory regions for the 18–59 age group. Abbreviations: HA, head airway; TR, tracheobronchial region; AR, alveolar region; EDI, the estimated daily intake; HQ, hazard quotient; DED, daily exposure dose; EQ, the number of equivalent cigarettes.

#### 3.4.2 Health Risks of EPFRs in PM_2.5_ through Inhalation Exposure

We determined the DEDs and EQs for EPFRs in atmospheric PM_2.5_ by age groups. The DEDs in the HA region varied from 0.19 × 10^12^ spins kg^–1^ bw day^–1^ to 0.60 × 10^12^ spins kg^–1^ bw day^–1^ for different age groups. The observed age distribution pattern of these DED was similar to that associated with CPs. Specifically, infants and children aged 6 months to 6 years, as well as young adults aged 18 to 59 years, exhibited higher DED values compared to other age groups. Peak DED values were observed in the 9-month to 4-year-old age group, ranging from 0.54 × 10^12^ spins kg^–1^ bw day^–1^ to 0.60 × 10^12^ spins kg^–1^ bw day^–1^. The DED values for the 45–59 and 18–44 years old groups were 0.52 × 10^12^ spins kg^–1^ bw day^–1^ and 0.49 × 10^12^ spins kg^–1^ bw day^–1^, respectively (Figure 4C, Table S21). These findings are consistent with a previous study in Harbin, which reported a similar age distribution of inhaled EPFR concentrations.^29^ Despite similar inhaled concentrations of EPFRs, the age distribution of EQ, calculated from median EPFR concentrations, differed significantly from the DED distribution. While DED were highest for young children, EQ values were significantly elevated in young to middle-aged adults (18–59 years old) compared to all other age groups (Figure 4D). This discrepancy is likely attributable to the EQ calculation not accounting for body weight standardization, rendering the results more sensitive to the breathing frequency. Remarkably, the estimated exposure risks for the 18–44 and 45–59 years old groups were equivalent to that associated with daily smoking, with corresponding EQ of 0.285 (HA), 0.030 (TB), and 0.063 (AR) (Figure 4E, Table S22). These findings suggest that adults may experience disproportionately higher inhalation risks from PM_2.5_-bound EPFRs compared with younger and older populations. This elevated risk may be related to factors such as higher ventilation rates, longer duration of outdoor exposure, and potentially differences in deposition efficiency in the respiratory tract.^29^ The health risks of smoking, including but not limited to lung cancer and coronary heart disease, are well-documented.^103^ Specifically, daily smoking of one cigarette increases lung cancer risk by 7.7%.^104^ Research has also established an analogy between ambient PM_2.5_ pollution exposure and cigarette consumption, where a PM_2.5_ concentration of 22 μg/m^3^ is roughly equivalent to smoking one cigarette per day.^105^ Our study indicates that EPFRs alone can contribute an estimated “smoking equivalent” of approximately 0.3 cigarettes per day. While this value may appear incremental, its cumulative effects and significant public health implications cannot be overlooked. Such quantitative assessments are crucial for enhancing public comprehension of environmental pollution’s health risks and provide a robust theoretical foundation for developing targeted environmental health interventions.

The inhalation risk assessment of CPs and EPFRs in ambient PM_2.5_ conducted in this study is subject to several uncertainties. First, sampling was confined to the heating season, a period characterized by elevated PM_2.5_ concentrations and lower temperatures that favor the partitioning of target pollutants into the particulate phase.^106^ Consequently, extrapolating these results to annual averages may lead to an overestimation of the potential health risks. Second, this cigarette-equivalent metric is uncertain because cigarette smoke contains other toxicants^107^ and the comparison is limited to free radical content, overlooking their potentially differing health effect pathways. Hence, it serves as a conservative, radical-based communication tool, not a surrogate for overall health outcomes, and overinterpretation is cautioned. Moreover, risk assessment methodologies inherently involve uncertainties and may be influenced by unmeasured confounding factors. Therefore, continuous environmental monitoring and the integration of epidemiological and toxicological studies are warranted to comprehensively elucidate the potential long-term health effects.^108^ Moreover, according to the *Chinese Exposure Factors Handbook*,^46^ physiological parameters vary among different genders and regions. Under the assumption of consistent pollutant concentrations, populations in northwestern China may exhibit higher inhalation exposure due to prolonged outdoor time, while males typically show greater exposure levels than females, owing to higher breathing rates. Future studies should therefore incorporate regional and gender-specific differences to enhance the representativeness and generalizability of the assessment results.

## 4 Conclusion and Implication

This study conducted continuous PM_2.5_ sampling in Shijiazhuang during the cold heating season and found extensive occurrence of CPs and EPFRs. Source apportionment revealed that vehicular emissions and industrial emissions were primary sources of CPs, while atmospheric oxidation and vehicular emissions were the primary source of EPFRs. These insights provide recommendations for targeted emission reduction strategies, such as safer manufacturing practices and emission control technologies. Additionally, our findings demonstrated significant correlations between the concentrations of CPs and EPFRs and multiple atmospheric pollutants as well as meteorological factors, indicating potential synergistic effects and complex environmental interactions that influence their behavior and fate. Future research should emphasize mechanistic investigations into their transport, transformation, and interactions within atmospheric systems to deepen our understanding of their environmental dynamics. Furthermore, we innovatively applied age-specific physiological parameters derived from multistage Chinese populations and a particle deposition model to estimate the inhalation exposure risk of CPs and EPFRs across the entire human lifespan. Our assessment revealed that children (<6 years) and adults (≥18 years) exhibited higher inhalation exposure levels to both CPs and EPFRs, whereas individuals aged 18–59 years exhibited the highest cigarette equivalent inhalation exposure to EPFRs. Although current risk estimates suggest some safety margins, the persistence of CPs and EPFRs likely leads to underestimation of long-term health risks, emphasizing the importance of ongoing, comprehensive health risk evaluations for public health protection.

## Supplementary Material



## Notes

Instrumental analysis methods, equations for quantifying CPs, and estimation methods for PM_2.5_ deposition flux and EPFRs-mediated ·OH production rate (Texts S1, S2, S4–S5, and S7); analytical quality control, robustness assessment of the PMF model, and validation data (Text S3; Tables S3–S12); quantitative ion information and carbon/chlorine congener group profiles of SCCPs, MCCPs, and LCCPs (Table S1, Figure S1); relationships between environmental factors and CPs/EPFRs concentrations, along with source apportionment (Figures S2–S4; Tables S16–S17); pollutant concentration data from the Shijiazhuang sampling site (Table S15); and health risk assessment parameters along with the EDI, HQ, MOE, DED, and EQ results for 17 age groups via PM_2.5_ inhalation (Tables S13–S14, S18–S22; Figure S5) (PDF)
