# Supplementary material for: Occurrence, Sources, and Health Risks of Chlorinated Paraffins and Environmental Persistent Free Radicals in Urban PM2.5 from the Megacity Shijiazhuang, China
Source: ACS Omega. 2026 Apr 27;11(18):26978–92. doi: 10.1021/acsomega.6c00507 (PMC13177017; doi:10.1021/acsomega.6c00507)
Supplement: Supplementary file 1 [file ao6c00507_si_001.pdf]

Supporting Information

Occurrence, sources, and health risks of chlorinated paraffins and environmental persistent free radicals in urban $PM_{2.5}$ from the megacity of Shijiazhuang, China

Shuling Duan[a,b††], Wenyan Yan[a,c††], Xu Yang[d], Fan Yang[d], Ke Fang[a,b], Juan Liu[a,b], Qiao Yao[a,e], Xiaoyan Dong[a], Na Li[a], Mengyao Wang[a,c], Jiajun Xiao[a,c], Xiaona Wang[a,f], Keke Long[a,g], Yibin Sun[h], Hongyang Cui[a], Qin wang[a], Yi Wan[h], Rong Zhang[i], Fobang Liu[d], Chao Wang[a,b*], Song Tang[a,b,c*]

[a] China CDC Key Laboratory of Environment and Population Health, National Institute of Environmental Health, Chinese Center for Disease Control and Prevention, Beijing 100021, China

[b] National Key Laboratory of Intelligent Tracking and Forecasting for Infection Diseases, Chinese Center for Disease Control and Prevention, Beijing 100021, China

[c] Center for Global Health, School of Public Health, Nanjing Medical University, Nanjing, Jiangsu 211166, China

[d] Department of Environmental Science and Engineering, School of Energy and Power Engineering, Xi'an Jiaotong University, Xi'an, Shanxi 710049, China

[e] College of Environmental Sciences and Engineering, Beijing Forestry University, Beijing 100083, China

[f] School of Public Health, Shandong University, Jinan, Shangdong 250061, China

[g] School of Public Health, China Medical University, Shenyang, Liaoning 110122, China

[h] College of Urban and Environmental Sciences, Peking University, Beijing 100871, China

[i] Department of Toxicology, Hebei Medical University, Shijiazhuan, Heibei 050017, China

* Corresponding author at No. 7 Panjiayuan Nanli, Chaoyang District, Beijing, 100021, China.

** Corresponding author at No. 7 Panjiayuan Nanli, Chaoyang District, Beijing, 100021, China. E-mail addresses: wangchao@nieh.chinacdc.cn (C. Wang), tangsong@nieh.chinacdc.cn (S. Tang).

Contents Pages: S1-S26, Texts: S1-S7, Figures: S1-S5, Tables: S1-S22

Contents

Supplemental Methods S1

Text S1. Instrument methods of CPs S1

Text S2. Equations to quantify CPs S2

Text S3. Quality control S3

Text S4. The calculation process for the deposition fluxes of $PM_{2.5}$ S4

Text S5. The analysis process for carbon fractions and elements in $PM_{2.5}$ S5

Text S6. Robustness and reliability assessment for positive matrix factorization (PMF) model S6

Text S7. Estimation of EPFRs-Mediated ·OH production rate S7

Supplemental Figures S8

Figure S1. Relative abundance of carbon and chlorine congener group profiles of CPs in $PM_{2.5}$.. ...S8

Figure S2. The relationships between environmental factors and concentrations of CPs S9

Figure S3. The relationships among environmental factors S10

Figure S4. The association of specific environmental factors with concentrations of CPs S11

Figure S5. EDIs of ΣSCCPs, ΣMCCPs and ΣLCCPs in $PM_{2.5}$ S12

Supplemental Tables S13

Table S1. Quantitative ions of SCCPs, MCCPs and LCCPs S133

Table S2. Regression equation between total response factor and calculated chlorine content, linear range and method detection limits S154

Table S3. Recovery rate and relative standard deviation of blank matix S165

Table S4. Comparison of Q(Robust) and Q(True) across different factor numbers S176

Table S5. Bootstrap factor mapping matrix for a two-factor solution. S16

Table S6. Bootstrap factor mapping matrix for a three-factor solution S246

Table S7. Bootstrap factor mapping matrix for a four-factor solution S256

Table S8. Bootstrap factor mapping matrix for a five-factor solution S16

Table S9. Bootstrap factor mapping matrix for a six-factor solution S17

Table S10. Bootstrap factor mapping matrix for a seven-factor solution S17

Table S11. BS-DISP robustness diagnostics summary for the five-factor solution S17

Table S12. DISP rotational perturbation diagnostics for the five-factor solution S17

Table S13. Parameter values for the deposition fluxes of $PM_{2.5.}$ S18

Table S14. Parameters for EDIs calculations. S19

Table S15. Concentrations of CPs and EPFRs in $PM_{2.5}$ from Shijiazhuang S20

Table S16. CPs in the atmosphere during cold seasons: analytical methods and recent advances ...S22

Table S17. EPFRs in the atmosphere during cold seasons: analytical methods and recent advances S23

Table S18. EDIs of SCCPs, MCCPs and LCCPs by age groups via inhalation of $PM_{2.5}$ S23

Table S19. HQ of SCCPs, MCCPs and LCCPs by age groups via inhalation of $PM_{2.5}$ S24

Table S20. MOE of SCCPs, MCCPs and LCCPs by age groups via inhalation of $PM_{2.5}$ S25

Table S21. DED of EPFRs by age groups via inhalation of $PM_{2.5}$ S276

Table S22. EQ of EPFRs by age groups via inhalation of $PM_{2.5}$ S287

Supplemental Methods

Text S1. Instrument methods of CPs

Chromatographic conditions: Column temperature was maintained at 40°C. The flow rate was 0.1 mL/min, and the injection volume was 3 μL. The autosampler temperature was set to 4℃. The mobile phase consisted of (A) water and (B) a 10 μM solution of Ph4PCl in methanol. A gradient elution program was employed as follows: 0-1 min, 10% B; 1-9 min, 10% to 100% B; 9-15 min, 100% B; 15-15.5 min, 100% to 30% B; 15.5-16.5 min, 30% to 10% B; and 16.5-20 min, 10% B.

Mass spectrometry conditions: Heated electrospray ionization (HESI) source in negative ionization mode was employed for detection. The ion transfer tube temperature was set to 200℃. The spray voltage was 2.5 kV. Sheath gas pressure was maintained at 35 psi, and the auxiliary gas flow rate was 3.5 L/min, with a corresponding temperature of 300℃. The sweep gas flow rate was 1 L/min. Full scan mode was used to acquire data over a range of 100–1000 m/z with a resolution of 140,000 FWHM. The automatic gain control (AGC) target ion number was 5×10^6, and the maximum injection time (IT) was 250 ms.

Text S2. Equations to quantify CPs

In this study, internal standard quantification was employed using 5 ng of Ph_4PCl as the internal standard. A series of standard solutions with calculated mass concentrations of 10, 20, 40, 80, 160, 320, and 1000 ng/mL were prepared. The relative total area, calculated chlorine content, and response factors for SCCPs, MCCPs, and LCCPs in these standards were determined. A regression equation was then established between the total response factor (y) and the calculated chlorine content (x). The chlorine content of the actual samples was then calculated, and the resulting value was substituted into the regression equation to obtain the total concentration of CPs in the samples[1]. The detailed calculation procedure is as follows:

1) The relative total area of each class of CPs in the standards was calculated separately for SCCPs, MCCPs, and LCCPs, where "i" represents the "i" homolog group within the CPs:

$$\text{Relative total CP area}=\sum_{i}\frac{\text{area i(congener group)}}{\text{area i(ISTD)}}\quad(1)$$

2) The total response factors for each class of CPs in the standards were calculated. Since the amount of internal standard added was the same in all standards and actual samples, the internal standard concentration could be eliminated from the equation:

$$\text{Total response factor(CP mixture Std)}=\frac{\text{rel. total CP area(Std.)}}{\text{amount CPs(Std.)}}\quad(2)$$

3) The chlorine content of each class of CPs was calculated for all standards and samples:

$$\text{Chlorinecontent(CP mixt.)}=\sum_{i}\frac{\text{rel.area(cong.groupi)}\times\text{chlorine content(cong.groupi)}\times\text{ chlorine mass}}{\text{rel. total CP area}\times\text{ atomic mass(cong.groupi)}}\quad(3)$$

4) Standard solutions of CPs with varying chlorine content were used to establish regression equations correlating total response factors of different CP congeners with calculated chlorine content：

$$\text{Total response facotr (CPs in the } \mathit{Std})=\mathit{ax}\ (\text{ch}\mathit{lorine\ content})+\mathit{b}\quad(4)$$

5) The calculated chlorine content of the actual sample was then substituted into the regression equation derived in 4) to obtain the total response factor of CPs in each sample. The concentration of CPs in the sample could then be calculated using the following equation:

$$\text{CP amount(sample)}=\frac{\text{relative total area(sample)}}{\text{total response factor(calculated for the sample)}}\quad(5)$$

Text S3. Quality control

To ensure the accuracy of the detection process, a series of blanks were established and assessed in conjunction with the actual samples, including both sampling blanks and procedural blanks. This approach allowed us to appropriately deduct these values during the measurements of the actual samples. The mass spectrometer was calibrated weekly using the pierce ion calibration solution to maintain a mass detection deviation of less than 2 ppm. The correlation coefficient (R^2) between the total response factor (y) and the calculated chlorine content (x) was greater than 0.96 (**Table S3**). To determine the method detection limits (MDLs) for CPs, we analyzed extracted matrix samples at concentrations ranging from 2 to 5 times the estimated MDLs, using seven replicates and employing a threefold S/N for calculations (**Table S3**). The average recovery rates of CPs at different concentration levels (2, 10, and 20 ng) ranged from 77.38% to 101.39%, with relative standard deviations ranging from 2.90% to 12.84% (**Table S2**).

Text S4. The calculation process for the deposition fluxes of $PM_{2.5}$

The International Commission on the Radiological Protection (ICRP) model demonstrated that particulate matter can enter three main regions of human respiratory tract (head airway (HA), tracheobronchial region (TB) and alveolar region (AR)) *via* particles in different size-ranges[2]. The deposition efficiency of particles into the head airway ($DF_{HA,i}$) is estimated by

$$DF_{HA,i}=IF_i\times(1/(1+\exp(6.84+1.183\ln D_{Pi}))+1/(1+\exp(0.924-1.185\ln D_{Pi})))$$

where D_{Pi} (μm) is the mean diameter of $PM_{2.5}$. IF_i is the inhalable fraction of particles with size-range i, which is estimated by

$$IF_i=1-0.5\times(1-1/(1+0.00076\times D_{pi}^{2.8}))$$

The deposition efficiency of particles into the tracheobronchial region ($DF_{TR,i}$) is estimated by

$$DF_{TR,i}=0.00352/D_{Pi}\times$$
$$(\exp(-0.234\times(\ln D_{Pi}+3.40)^2)+63.9\times\exp(-0.819\times(\ln D_{Pi}-1.61)^2))$$

The deposition efficiency of particles into the alveolar region ($DF_{AR,i}$) is estimated by

$$DF_{AR,i}=0.0155/D_{Pi}\times$$
$$(\exp(-0.416\times(\ln D_{Pi}+2.84)^2)+11.91\times\exp(-0.482\times(\ln D_{Pi}-1.362)^2))$$

Given that $PM_{2.5}$ refers to particulate matter with an aerodynamic diameter less than or equal to 2.5 μm, the deposition efficiency of $PM_{2.5}$ was calculated by integrating the deposition efficiency function *DF*i over the range of particle diameters from 0 to 2.5 μm. As presented in **Table S16**.

Text S5. The analysis process for carbon fractions and elements in $PM_{2.5}$

The analysis of organic carbon (OC) and elemental carbon (EC) in each $PM_{2.5}$ sample was performed using an OC/EC analyzer (Sunset Laboratory Model 5L, USA) following the improve thermal-optical reflectance protocol. A 4.5 cm^2 punch of each sample was placed in a quartz furnace and analyzed. Quantification of OC1-OC4 and EC1-EC3 was conducted using a calibration curve established with sucrose (Macklin, China) as the standard.

A total of 12 elements (Mg, Al, Cr, Mn, Fe, Ni, Cu, Zn, As, Cd, Pb, Se) were determined using a NexION 300X inductively coupled plasma mass spectrometer (ICP-MS, PerkinElmer, USA). A 9 cm^2 section of each filter was digested in a quartz tube with a mixture of 6 mL HNO_3 (Beijing Chemical Works, China), 1 mL HCl (ANPEL, China), and 1 mL HF (ANPEL, China) *via* microwave digestion for 30 minutes and then evaporated to near-dryness using an acid evaporation system. Quantification was performed using a calibration curve prepared with elemental standard solutions (NCS, China), and rhenium was used as an internal standard to correct for signal drift.

Text S6. Robustness and reliability assessment for positive matrix factorization (PMF) model

To evaluate the robustness and reliability of PMF model, we employed several criteria. First, the ratio of Q (robust) to Q (true) was examined; for the current dataset with a five-factor solution, this ratio was 0.93, close to 1, indicating a near-optimal fit[3]. Additionally, a bootstrap resampling method with 20 iterations was employed, resulting in a mapping stability of 95% to 100% for the five-factor model without any displacement run exchanges, supporting the appropriateness of the selected number of factors[4](**Tables S7-S15)**. Second, the S/N of each indicator was considered; all included indicators exhibited S/N values greater than 5, demonstrating strong and reliable signals. Since the total CPs value was derived from the sum of three types of CP values, it was designated as a "weak" indicator within the model. Accordingly, to minimize the influence of the $PM_{2.5}$ variable on the model, $PM_{2.5}$ was also assigned as a "weak" indicator[5]. Furthermore, the scaled residuals for all indicators fell within the acceptable range of -3 to 3, further confirming the good fit of the model[6].

Text S7. Estimation of EPFRs-Mediated ·OH Production Rate

Data Sources: EPFRs catalytic efficiency: Each EPFRs can catalytically produce approximately 10 ·OH radicals over its lifetime[7]. EPFRs concentration in $PM_{2.5}$ from this study: 1.03×10^{12} to 6.11×10^{13} spins/m^3. Measured atmospheric half-life of EPFRss: 43.5–63.0 days[8]. A representative lifetime of 50 days is used for this calculation.

$$k_{EPFR}=\frac{1}{\text{lifetime}}=\frac{1}{50\times24\times3600}=2.31\times10^{-7}s^{-1}$$

Calculation of Time-Averaged ·OH Production Rate from EPFRss ($R_{\cdot OH,\ EPFRs}$)

Assuming EPFRss release 10 ·OH radicals at a constant rate throughout their atmospheric lifetime:

$$R_{\cdot OH,EPFR}=C_{EPFR}\times E_{EPFR}\times k_{EPFR}$$

Using the highest measured EPFRs concentration from this study ($C_{EPFRs}=6.11\times10^{13}$ spins/m^3) to represent a scenario most favorable for EPFRs contribution:

$$R_{\cdot OH,EPFR}=(6.11\times10^{13)}\times10\times(2.31\times10^{-7})=1.41\times10^{2}\cdot OH/cm^{3}/s$$

Contribution of EPFRss to the Steady-State Atmospheric ·OH Concentration

The steady-state concentration of atmospheric ·OH ($[\cdot OH]_{ss}$) is determined by the balance between its production and loss. The major sink for ·OH is reaction with trace gases such as CH_4 and CO, with a total pseudo-first-order loss rate constant (k_{loss}) of approximately 1 s^{-1} (a typical value). Therefore, the incremental contribution of EPFRss to the steady-state ·OH concentration $\Delta[\cdot OH]_{EPFRs}$ is:

$$\Delta[\cdot OH]_{EPFR}=\frac{R_{\cdot OH,EPFR}}{k_{loss}}=1.41\times10^{2}\ \text{molecules/cm}^{3}$$

Background ·OH concentration used in this section: $[\cdot OH]_{bg}=0.5\times10^{6}$ molecules/cm^3

Fractional Contribution of EPFRss:

$$\frac{\Delta[\cdot OH]_{EPFR}}{[\cdot OH]_{bg}}=0.028\%$$

Even under the most favorable assumptions (maximum EPFRs concentration, 10 ·OH per EPFRs, and a low-end ·OH loss rate), the contribution of EPFRs to the background ·OH concentration is far less than 0.03%.

Supplemental Figures

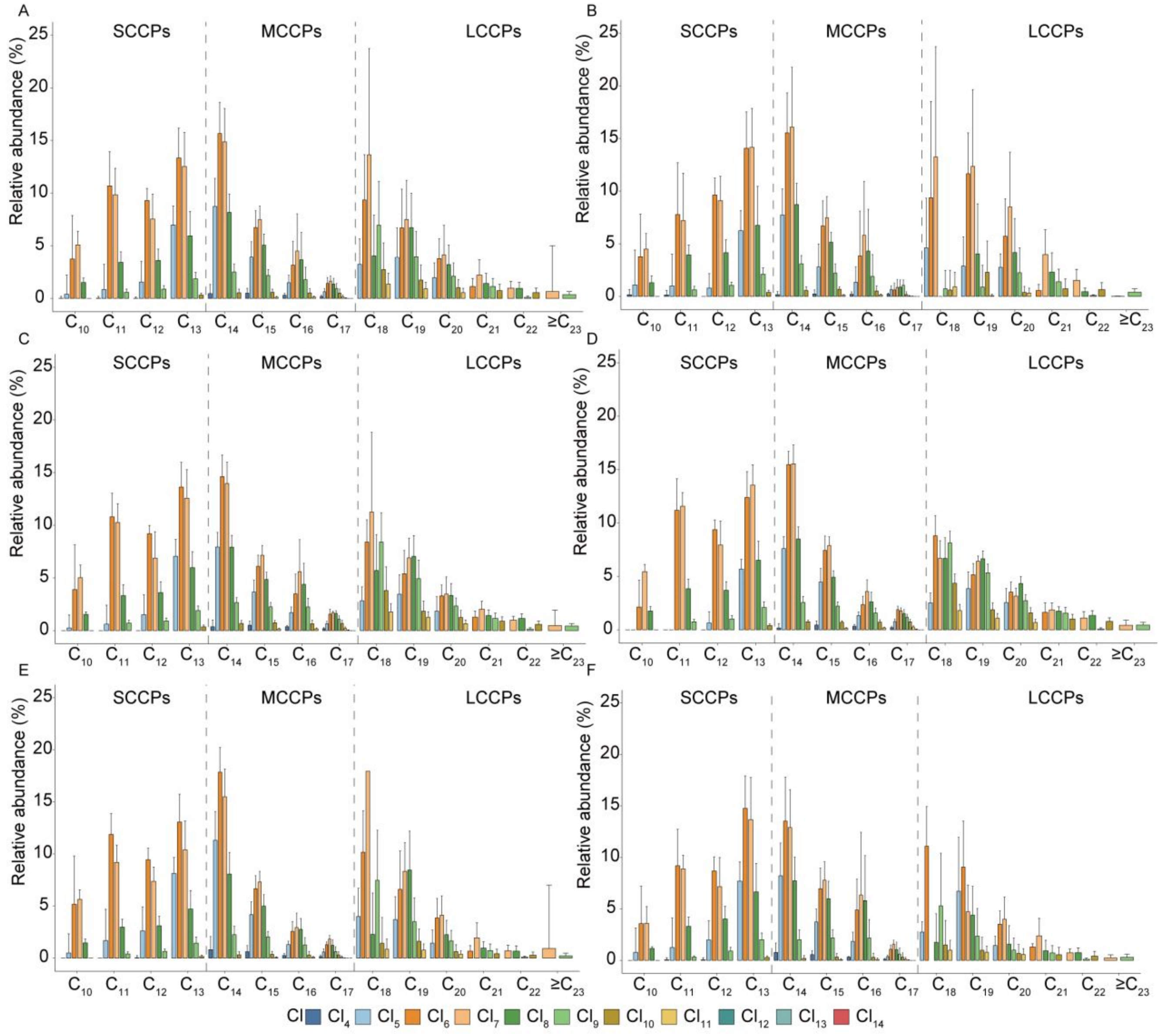

Figure S1. Relative abundance of carbon and chlorine congener group profiles of SCCPs, MCCPs, and LCCPs in $PM_{2.5}$ in all months (**A**), November (**B**), December (**C**), January (**D**), February (**E**), and March (**F**) from Shijiazhuang.

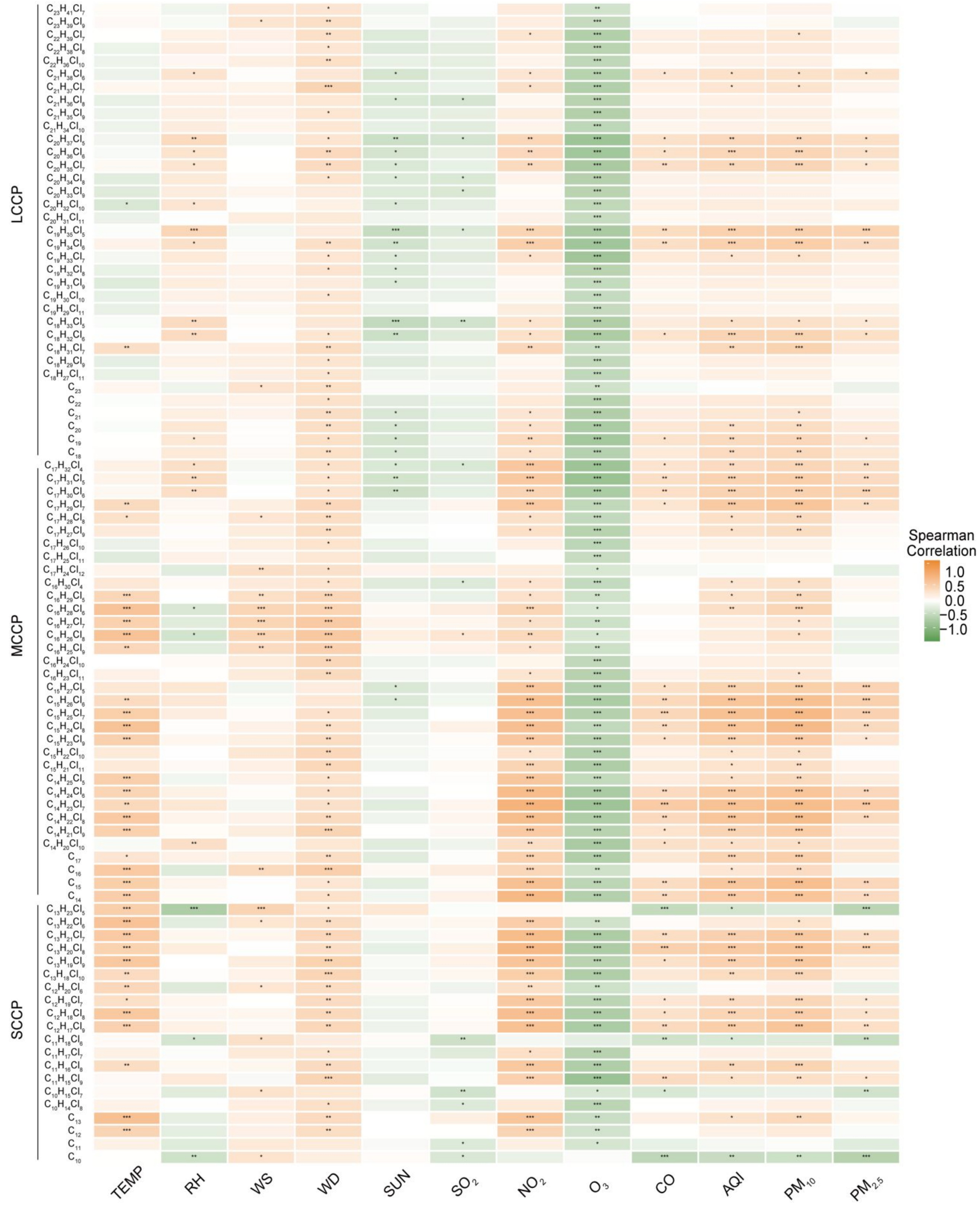

Figure S2. Spearman's correlation heatmap showing the relationships between environmental factors and concentrations of frequently detected CPs (detection rate > 30%) in $PM_{2.5}$. Asterisks denote statistical significance: *$p < 0.05$, **$p < 0.01$, and ***$p < 0.001$.

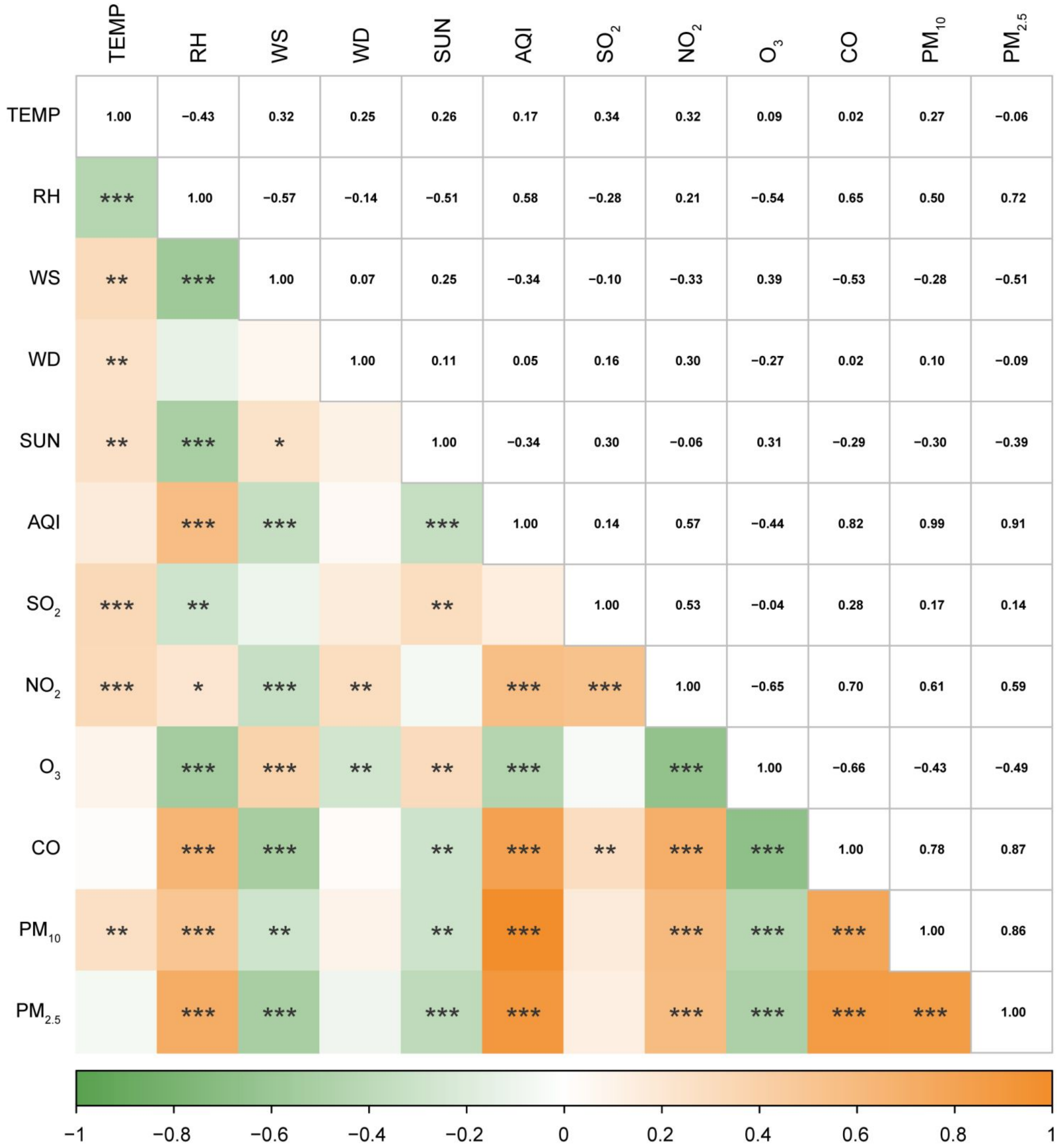

Figure S3. Spearman's correlation heatmap showing the relationships among environmental factors. Asterisks denote statistical significance: *$p < 0.05$, **$p < 0.01$, and ***$p < 0.001$.

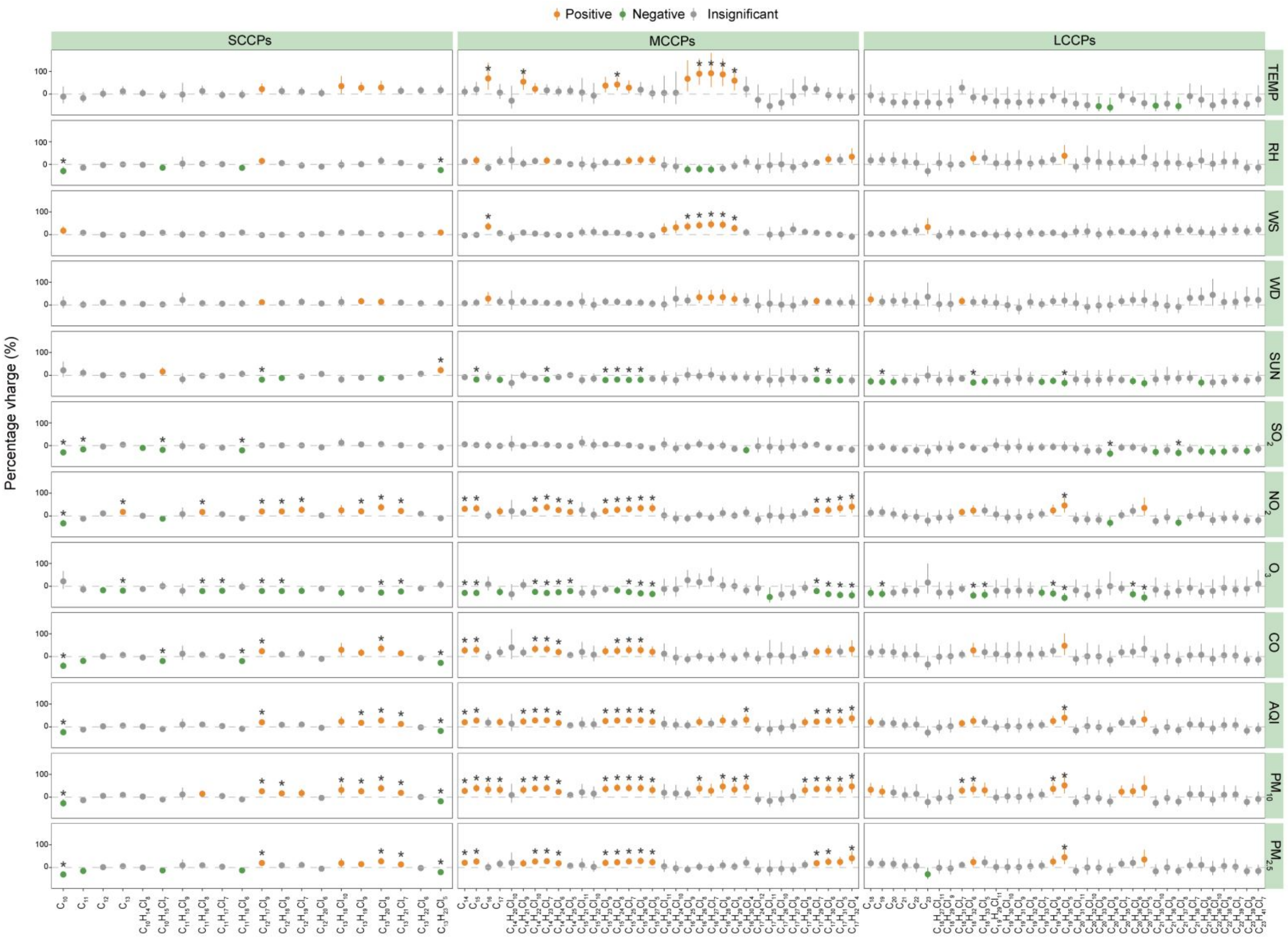

Figure S4. Linear mixed effects model demonstrating the association of specific environmental factors with concentrations of frequently detected CPs (detection rate > 30%). Asterisks denote statistical significance: *$p < 0.05$.

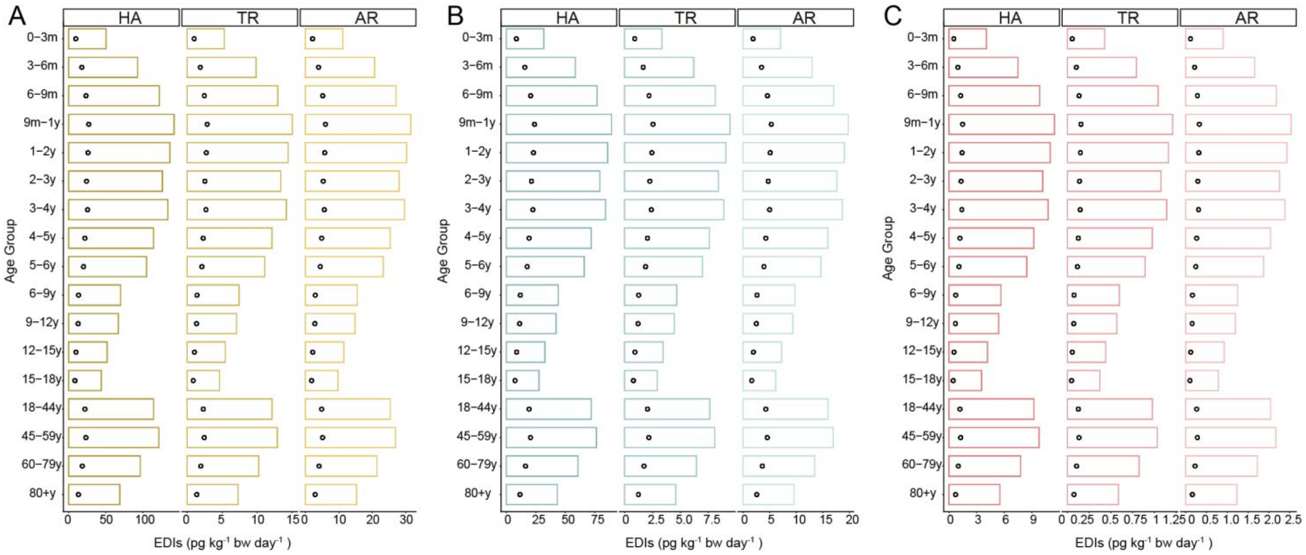

Figure S5. The estimated daily intakes (EDIs) of ΣSCCPs (**A**), ΣMCCPs (**B**), and ΣLCCPs (**C**) in $PM_{2.5}$ deposited in the human respiratory tract (HA, TB and AR) across different age groups. Circles within the bar indicate the median values, and the tops of the bars represent the maximum values.

Supplemental Tables

Table S1. Quantitative ions of SCCPs, MCCPs and LCCPs.

SCCPs	Quantification ion	MCCPs	Quantification ion	LCCPs	Quantification ion
$C_{10}H_{18}Cl_4$	314.98162	$C_{14}H_{26}Cl_4$	371.04422	$C_{18}H_{33}Cl_5$	461.06784
$C_{10}H_{17}Cl_5$	348.94264	$C_{14}H_{25}Cl_5$	405.00524	$C_{18}H_{32}Cl_6$	495.02887
$C_{10}H_{16}Cl_6$	382.90367	$C_{14}H_{24}Cl_6$	438.96627	$C_{18}H_{31}Cl_7$	530.98695
$C_{10}H_{15}Cl_7$	418.86175	$C_{14}H_{23}Cl_7$	474.92435	$C_{18}H_{30}Cl_8$	564.94798
$C_{10}H_{14}Cl_8$	452.82278	$C_{14}H_{22}Cl_8$	508.88538	$C_{18}H_{29}Cl_9$	598.909
$C_{10}H_{13}Cl_9$	486.7838	$C_{14}H_{21}Cl_9$	542.8464	$C_{18}H_{28}Cl_{10}$	632.87003
$C_{11}H_{20}Cl_4$	328.99727	$C_{14}H_{20}Cl_{10}$	576.80743	$C_{18}H_{27}Cl_{11}$	668.82811
$C_{11}H_{19}Cl_5$	362.95829	$C_{15}H_{28}Cl_4$	385.05987	$C_{19}H_{35}Cl_5$	475.08349
$C_{11}H_{18}Cl_6$	396.91932	$C_{15}H_{27}Cl_5$	419.02089	$C_{19}H_{34}Cl_6$	509.04452
$C_{11}H_{17}Cl_7$	432.8774	$C_{15}H_{26}Cl_6$	452.98192	$C_{19}H_{33}Cl_7$	545.0026
$C_{11}H_{16}Cl_8$	466.83843	$C_{15}H_{25}Cl_7$	488.94	$C_{19}H_{32}Cl_8$	578.96363
$C_{11}H_{15}Cl_9$	500.79945	$C_{15}H_{24}Cl_8$	522.90103	$C_{19}H_{31}Cl_9$	612.92465
$C_{12}H_{22}Cl_4$	343.01292	$C_{15}H_{23}Cl_9$	556.86205	$C_{19}H_{30}Cl_{10}$	646.88568
$C_{12}H_{21}Cl_5$	376.97394	$C_{15}H_{22}Cl_{10}$	590.82308	$C_{19}H_{29}Cl_{11}$	682.84376
$C_{12}H_{20}Cl_6$	410.93497	$C_{15}H_{21}Cl_{11}$	626.78116	$C_{20}H_{37}Cl_5$	489.09914
$C_{12}H_{19}Cl_7$	446.89305	$C_{16}H_{30}Cl_4$	399.07552	$C_{20}H_{36}Cl_6$	523.06017
$C_{12}H_{18}Cl_8$	480.85408	$C_{16}H_{29}Cl_5$	433.03654	$C_{20}H_{35}Cl_7$	559.01825
$C_{12}H_{17}Cl_9$	514.8151	$C_{16}H_{28}Cl_6$	466.99757	$C_{20}H_{34}Cl_8$	592.97928
$C_{13}H_{23}Cl_5$	390.98959	$C_{16}H_{27}Cl_7$	502.95565	$C_{20}H_{33}Cl_9$	626.9403
$C_{13}H_{22}Cl_6$	424.95062	$C_{16}H_{26}Cl_8$	536.91668	$C_{20}H_{32}Cl_{10}$	660.90133
$C_{13}H_{21}Cl_7$	460.9087	$C_{16}H_{25}Cl_9$	570.8777	$C_{20}H_{31}Cl_{11}$	696.85941
$C_{13}H_{20}Cl_8$	494.86973	$C_{16}H_{24}Cl_{10}$	604.83873	$C_{21}H_{38}Cl_6$	537.07582
$C_{13}H_{19}Cl_9$	528.83075	$C_{16}H_{23}Cl_{11}$	640.79681	$C_{21}H_{37}Cl_7$	573.0339
$C_{13}H_{18}Cl_{10}$	562.79178	$C_{17}H_{32}Cl_4$	413.09117	$C_{21}H_{36}Cl_8$	606.99493
-	-	$C_{17}H_{31}Cl_5$	447.05219	$C_{21}H_{35}Cl_9$	640.95595
-	-	$C_{17}H_{30}Cl_6$	481.01322	$C_{21}H_{34}Cl_{10}$	674.91698
-	-	$C_{17}H_{29}Cl_7$	516.9713	$C_{22}H_{39}Cl_7$	587.04955
-	-	$C_{17}H_{28}Cl_8$	550.93233	$C_{22}H_{38}Cl_8$	621.01058
-	-	$C_{17}H_{27}Cl_9$	584.89335	$C_{22}H_{37}Cl_9$	654.9716
-	-	$C_{17}H_{26}Cl_{10}$	618.85438	$C_{22}H_{36}Cl_{10}$	688.93263
-	-	$C_{17}H_{25}Cl_{11}$	654.81246	$C_{23}H_{41}Cl_7$	601.0652
-	-	$C_{17}H_{24}Cl_{12}$	688.77349	$C_{23}H_{40}Cl_8$	635.02623
-	-	$C_{17}H_{23}Cl_{13}$	722.73451	$C_{23}H_{39}Cl_9$	668.98725
-	-	$C_{17}H_{22}Cl_{14}$	756.69554	$C_{24}H_{42}Cl_8$	649.04188
-	-	-	-	$C_{25}H_{45}Cl_7$	629.0965

Table S2. Regression equation between total response factor and calculated chlorine content, linear range and method detection limits.

Target Compound	Ionization Mode	Regression Equation	Regression Equation	R^2	MDLs (ng/m^3)
∑SCCPs	$[M+Cl]^-$	10.0~1000.0	y=96.70x-43.55	0.961	0.29
∑MCCPs		10.0~1000.0	y=138.25x-58.57	0.971	0.03
∑LCCPs		10.0~1000.0	y=177.19x-71.02	0.999	0.01

MDLs: the method detection limits.

Table S3. Recovery rate and relative standard deviation of blank matrix (n=6).

Target Compound	2 ng		10 ng		20 ng	
	Recovery rate (%)	RSD (%)	Recovery rate (%)	RSD (%)	Recovery rate (%)	RSD (%)
∑SCCPs	81.64	7.07	93.11	7.79	95.04	11.21
∑MCCPs	77.38	7.01	94.31	11.21	87.41	2.9
∑LCCPs	80.79	12.84	99.78	7.99	101.39	6.35

RSD: the relative standard deviation.

Table S4. Comparison of Q(Robust) and Q(True) across different factor numbers

The number of factors	Q(Robust)	Q(True)	The ratio of Q (robust) to Q (true)
2	8416.36	10265	0.819908427
3	6863.04	8658.91	0.792598607
4	5245.78	6049.73	0.867109772
5	4018.74	4351.67	0.923493739
6	3124.68	3325.37	0.939648821
7	2389.73	2545.01	0.938986487

Table S5. Bootstrap factor mapping matrix for a two-factor solution

Mapping of bootstrap factors to base factors:	Factor 1	Factor 2	Unmapped
Boot Factor 1	100	0	0
Boot Factor 2	0	100	0

Table S6. Bootstrap factor mapping matrix for a three-factor solution

Mapping of bootstrap factors to base factors:	Factor 1	Factor 2	Factor 3	Unmapped
Boot Factor 1	100	0	0	0
Boot Factor 2	0	100	0	0
Boot Factor 3	3	13	82	2

Table S7. Bootstrap factor mapping matrix for a four-factor solution

Mapping of bootstrap factors to base factors:	Factor 1	Factor 2	Factor 3	Factor 4	Unmapped
Boot Factor 1	100	0	0	0	0
Boot Factor 2	1	83	3	11	2
Boot Factor 3	2	0	90	7	1
Boot Factor 4	0	0	0	100	0

Table S8. Bootstrap factor mapping matrix for a five-factor solution

Mapping of bootstrap factors to base factors:	Factor 1	Factor 2	Factor 3	Factor 4	Factor 5	Unmapped
Boot Factor 1	97	0	1	0	2	0
Boot Factor 2	1	96	0	3	0	0
Boot Factor 3	0	1	97	1	1	0
Boot Factor 4	1	0	0	98	1	0
Boot Factor 5	0	0	0	0	100	0

Table S9. Bootstrap factor mapping matrix for a six-factor solution

Mapping of bootstrap factors to base factors:	Factor 2	Factor 3	Factor 4	Factor 5	Factor 6	Unmapped
Boot Factor 1	1	1	2	1	6	0
Boot Factor 2	70	3	4	1	6	0
Boot Factor 3	0	100	0	0	0	0
Boot Factor 4	0	0	100	0	0	0
Boot Factor 5	0	0	1	98	0	0
Boot Factor 6	2	0	3	1	91	0

Table S10. Bootstrap factor mapping matrix for a seven-factor solution

Mapping of bootstrap factors to base factors:	Factor 1	Factor 2	Factor 3	Factor 4	Factor 5	Factor 6	Factor 7	Unmapped
Boot Factor 1	92	3	3	1	0	0	1	0
Boot Factor 2	0	96	1	1	0	0	2	0
Boot Factor 3	1	3	91	0	1	0	4	0
Boot Factor 4	0	5	0	87	4	0	3	1
Boot Factor 5	0	4	2	1	93	0	0	0
Boot Factor 6	0	0	0	0	0	100	0	0
Boot Factor 7	0	0	0	0	0	0	100	0

Table S11. BS-DISP robustness diagnostics summary for the five-factor solution

BS-DISP Diagnostics	
# of Cases Accepted	99
% of Cases Accepted	99%
Largest Decrease in Q	-21.62299919
%dQ	-0.5430172
# of Decreases in Q	1
# of Swaps in Best Fit	0
# of Swaps in DISP	0
Swaps by Factor	0

Table S12. DISP rotational perturbation diagnostics for the five-factor solution

DISP Diagnostics	
Error Code	0
Largest Decrease in Q	-0.085000001
%dQ	-0.0021346
Swaps by Factor	0

Table S13. Parameter values for the deposition fluxes of $PM_{2.5.}$

Diameter	DF_{HA}	DF_{TR}	DF_{AR}
≤2.5μm	0.898	0.095	0.199

Table S14. Parameters for EDIs calculations.

Age	BW (kg)	IR (m^3/d)	EF
0−3 m	6.4	3.7	0.035
3−6 m	7.9	4.7	0.063
6−9 m	9.1	5.4	0.083
9 m−1 y	9.8	5.9	0.095
1−2 y	11.2	5.7	0.108
2−3 y	13.5	6.3	0.109
3−4 y	15.6	8.0	0.105
4−5 y	17.7	8.4	0.097
5−6 y	19.6	8.8	0.094
6−9 y	26.5	10.1	0.074
9−12 y	36.8	13.2	0.075
12−15 y	47.3	13.5	0.073
15−18 y	54.8	14.0	0.069
18−44 y	61.9	16.7	0.171
45−59 y	63.5	16.7	0.186
60−79 y	60.3	13.8	0.170
80 y+	55.5	12.0	0.128

(1) BW: body weight;

(2) IR: the inhalation rate;

(3) EF: the outdoor exposure fraction.

All the values were cited from the Exposure Factors Handbook of Chinese Population(Adults)[9] and the Highlights of the Chinese Exposure Factors Handbook (Children)[10].

Table S15. Concentrations of CPs and EPFRs in $PM_{2.5}$ from Shijiazhuang (n=104).

Category	DF[a] (%)	Min	P_{25}	Median	Mean	P_{75}	Max	SD
SCCPs								
Cl (%)	-	57.25	58.05	58.44	58.40	58.82	59.43	-
C_{10}	100	3.22	26.49	51.95	71.26	87.13	487.42	79.41
C_{11}	100	23.59	84.33	131.82	156.19	182.28	882.64	113.33
C_{12}	100	36.19	86.48	117.66	135.39	165.40	580.46	78.40
C_{13}	100	77.55	160.50	215.34	235.87	290.32	863.60	126.81
∑SCCPs	100	159.53	385.74	526.84	598.71	701.39	2763.53	363.18
MCCPs								
Cl (%)	-	51.94	53.37	53.72	53.68	54.19	54.75	-
C_{14}	100	59.02	166.45	235.46	255.55	324.71	810.96	132.01
C_{15}	100	20.89	83.37	125.45	139.15	189.72	401.31	78.57
C_{16}	100	8.82	41.10	61.45	99.43	95.80	1266.38	167.98
C_{17}	100	3.41	16.64	32.63	36.77	51.46	136.66	24.39
∑MCCPs	100	92.14	323.51	466.58	530.90	651.88	1737.13	321.83
LCCPs								
Cl (%)	-	46.42	49.50	50.75	51.23	51.91	59.72	-
C_{18}	97.12	-	4.30	12.94	14.78	21.33	64.53	12.41
C_{19}	100	0.79	3.43	8.62	11.60	16.31	58.04	11.03
C_{20}	100	0.24	1.56	4.95	6.80	8.73	54.44	7.77
C_{21}	100	0.04	0.62	1.97	2.96	3.93	35.28	4.14
C_{22}	100	0.01	0.15	0.82	1.24	1.57	16.06	1.87
C_{23}	100	-	0.03	0.17	0.38	0.49	7.80	0.83
C_{24}	-	-	-	-	-	-	-	-
C_{25}	8.65	-	-	-	0.11	-	7.87	0.84
∑LCCPs	100	2.05	10.82	28.74	37.85	53.73	222.42	36.45
[b]Total CPs	100	347.76	817.89	1006.62	1167.46	1414.60	3886.56	656.13
[c]EPFRs×10^{12}	100	1.03	7.97	11.75	14.34	19.22	61.12	9.66

a: DF represents detection frequency; b: the unit of CPs is pg/m^3; c: the unit of EPFRs is spins/m^3

Table S16. CPs in the atmosphere during cold seasons: analytical methods and recent advances.

Sampling Time	Country	Sample Type	Sampler	Analytical Instrument	SCCP (pg/m^3)				MCCP (pg/m^3)				LCCP (pg/m^3)				Ref
					mean	min	max	Median	mean	min	max	Median	mean	min	max	Median	
2006 (winter)	India	Air	PUF−PAS	GC-ECNI-LRMS	10200	-	47400	-	3620	-	38200	-	-	-	-	-	11
2011 (winter)	Pakistan	Air	PUF−PAS	GC-ECNI-LRMS	5130	-	47400	-	4210	-	38200	-	-	-	-	-	11
2020 (winter)	Pakistan	Air	PUF-PAS	APCI-MS	-	-	2900	1100	-	-	1100	200	-	-	-	-	12
2013 (Spring)	Zurich, Switzerland	Air	PUF−PAS	GC-ECNI-HRMS	6400	1100	42000	-	2700	-	-	-	-	-	-	-	13
2013-2015 (Winter)	Brisbane, Australia	Gaseous plus particulate phases	PUF−PAS	TOF-HRMS	31-180	-	1700	-	26-160		930		42 - 97	-	-	-	14
2013-2015 (Winter)	Hanoi, Vietnam	Gaseous plus particulate phases	PUF−PAS	TOF-HRMS	140-3500	-	-	-	26 to 310	-	-	-	42	-	-	-	14
2014-2018 (winter)	West Antarctica	Particle phases	PUF−PAS	GC-QTOF	105	3.48	533	-	1.6	<0.26	6.28	-	-	-	-	-	15
2017-2018 (winter)	Toronto, Canada	Air	PUF−PAS	GC-ECNI-LRMS	25600	2960	66200	-	-	-	-	-	-	-	-	-	16
2020-2021 (winter)	Athens, Greece	Particle phases	PUF−PAS	UPLC-ESI-MS	3278	-	-	-	1394	-	-	-	-	-	-	-	17
2020 (winter)	Norway	Air	Active air sampler	GC-Orbitrap-MS	88.7	-	384	-	-	-	-	-	-	-	-	-	18
2011 (winter)	Beijing, China	Air	High volume sampler	GC-ECNI-LRMS	7700	1900	332000	-	-	-	-	-	-	-	-	-	19
2013-2014 (winter)	2 cities, China	Air	HV-1001F active air samplers	UPLC-QTOFMS	6112	-	-	-	4312	-	-	-	2991	-	-	-	20
2013-2015 (winter)	10 cities. China	$PM_{2.5}$	High volume sampler	HRGC-ECNI-LRMS	19900	1980	274000	-	15600	1270	312000	-	-	-	-	-	21
2016 (winter)	Jinan, China	$PM_{2.5}$	Medium volume samplers (TH16A)	GC-ECNI-LRMS	-	27000	105000	54800	-	-	-	-	-	-	-	-	22
2018 (winter)	Pearl River Delta, China	$PM_{2.5}$	Hi-vol (QFF)	LC-ESI-HRMS	7573	832	15900	6530	7108	1020	15800	6260	1952	369	13000	1410	23
2022 (autumn, winter)	Beijing, China	Particle phases	PUF−PAS	GC-ENCI-MS	56000	28000	108000	-	85000	16000	216000	-	-	-	-	-	24
2022-2023 (winter)	Shijiazhuang, China	$PM_{2.5}$	High volume sampler	UPLC-orbitrap-MS	599	160	2764	527	531	92	1737	467	38	2	222	28.74	This study

Table S17. EPFRs in the atmosphere during cold seasons: analytical methods and recent advances.

Sampling Time	Location	Sample Type	Sampler	Analytical Instrument	EPFRm (spins/g)			EPFRm (spins/m^3)			g factor	Ref
					mean	Min	Max	mean	Min	Max		
2019 (winter)	Zhengzhou, China	$PM_{2.5}$	Medium volume sampler	EPR spectrometer				2.72×10^{15}	1.63×10^{15}	1.07×10^{16}	2.0035 - 2.0037	[25]
2022 (winter)	Xiamen, China	PM	Derson stage eight sampler	EPR spectrometer				1.69×10^{14}	6.26×10^{13}	4.22×10^{14}	2.00434	[26]
2018-2019 (winter)	Dalian, China	$PM_{2.5}$	Low-volume sampler	Bruker EXM A-200 spectrometer				2.41×10^{15}			2.00329	[27]
2020 (winter)	Xi'an, China	$PM_{2.5}$	High-volume sampler	EPR spectrometer				3.17×10^{14}			2.0032	[28]
2020 (winter)	Beijing, China	$PM_{2.5}$	Medium-volume sampler	EPR spectrometer				1.42×10^{13}			2.00304	[29]
2020 (winter)	Yuncheng, China	$PM_{2.5}$	Medium-volume sampler	EPR spectrometer				2.82×10^{15}			2.00303	[29]
2020 (winter)	Zhoukou, China	Road dust	300 W handheld cordless vacuum	EPR spectrometer				8.43×10^{18}	2.24×10^{17}	3.72×10^{19}	2.0032-2.0039	[30]
2021 (winter)	Chengde, China	Road dust	Vacuum cleaner	EPR spectrometer	2.74×10^{18}	6.01×10^{17}	5.0×10^{17}				2.0029	[31]
2017 (winter)	Xuanwei, China	$PM_{1.1}$	High-volume air sampler	Electron spin resonance spectroscopy	2.16×10^{17}	1.44×10^{17}	4.26×10^{17}	7.03×10^{15}	2.86×10^{15}	1.79×10^{16}	2.0042	[32]
2022-2023 (winter)	Quzhou, China	PM	High-volume sampler	EPR spectrometer	1.14×10^{16}			4.49×10^{12}			2.0035	[33]
2021 (winter)	Ulaanbaatar, Mongolia	$PM_{2.5}$	high-volume sampler	EPR spectrometer				8.87×10^{13}			2.00288	[34]
2016 (spring)	Baton Rouge, USA	PM	fresh leaves	EPR spectrometer	6.94×10^{17}						2.0030-2.0050	[35]
2019 (winter)	Lahore, Pakistan	$PM_{2.5}$	air volume sampler	EPR spectrometer	2.3×10^{17}	9.3×10^{16}	4.7×10^{17}	1.2×10^{14}	2.9×10^{13}	2.9×10^{14}	2.003	[36]
2022-2023 (winter)	Shijiazhuang, China	$PM_{2.5}$	High volume sampler	EPR spectrometer	-	-	-	1.43×10^{13}	1.03×10^{12}	6.12×10^{13}	2.0036	This study

Table S18. EDIs (pg kg^{-1} bw day^{-1}) of SCCPs, MCCPs and LCCPs by age groups *via* inhalation of $PM_{2.5}$.

Age	SCCPs	MCCPs	LCCPs
0−3 m	10.66	9.44	0.58
3−6 m	19.75	17.49	1.08
6−9 m	25.95	22.98	1.42
9 m−1 y	30.13	26.69	1.64
1−2 y	28.96	25.65	1.58
2−3 y	26.80	23.73	1.46
3−4 y	28.37	25.12	1.55
4−5 y	24.25	21.48	1.32
5−6 y	22.23	19.69	1.21
6−9 y	14.86	13.16	0.81
9−12 y	14.17	12.55	0.77
12−15 y	10.98	9.72	0.60
15−18 y	9.29	8.22	0.51
18−44 y	24.31	21.53	1.33
45−59 y	25.77	22.82	1.41
60−79 y	20.50	18.15	1.12
80 y+	14.58	12.91	0.80

Table S19. HQ of SCCPs, MCCPs and LCCPs by age groups *via* inhalation of $PM_{2.5}$.

Age	SCCPs	MCCPs	LCCPs
0−3 m	1.07E-10	9.44E-11	5.81E-12
3−6 m	1.97E-10	1.75E-10	1.08E-11
6−9 m	2.59E-10	2.30E-10	1.42E-11
9 m−1 y	3.01E-10	2.67E-10	1.64E-11
1−2 y	2.90E-10	2.56E-10	1.58E-11
2−3 y	2.68E-10	2.37E-10	1.46E-11
3−4 y	2.84E-10	2.51E-10	1.55E-11
4−5 y	2.43E-10	2.15E-10	1.32E-11
5−6 y	2.22E-10	1.97E-10	1.21E-11
6−9 y	1.49E-10	1.32E-10	8.10E-12
9−12 y	1.42E-10	1.26E-10	7.73E-12
12−15 y	1.10E-10	9.72E-11	5.99E-12
15−18 y	9.29E-11	8.22E-11	5.07E-12
18−44 y	2.43E-10	2.15E-10	1.33E-11
45−59 y	2.58E-10	2.28E-10	1.41E-11
60−79 y	2.05E-10	1.82E-10	1.12E-11
80 y+	1.46E-10	1.29E-10	7.95E-12

Table S20. MOE of SCCPs, MCCPs and LCCPs by age groups *via* inhalation of $PM_{2.5}$.

Age	SCCPs	MCCPs	LCCPs
0−3 m	938067.85	2436194.03	171989959.21
3−6 m	506422.19	1315195.62	92849928.11
6−9 m	385383.92	1000855.11	70658176.96
9 m−1 y	331874.97	861890.57	60847585.09
1−2 y	345337.27	896852.62	63315829.28
2−3 y	373156.29	969099.55	68416304.02
3−4 y	352508.31	915476.04	64630601.86
4−5 y	412331.29	1070838.35	75598840.40
5−6 y	449748.36	1168011.76	82459070.22
6−9 y	673002.73	1747810.92	123391620.64
9−12 y	705564.41	1832374.73	129361639.78
12−15 y	911021.35	2365953.37	167031122.63
15−18 y	1076781.23	2796437.40	197422351.28
18−44 y	411435.83	1068512.82	75434663.16
45−59 y	388032.73	1007734.16	71143822.84
60−79 y	487880.37	1267041.89	89450380.50
80 y+	685844.74	1781162.06	125746137.93

Table S21. DED (× 10^{12} spins kg^{-1} bw day^{-1}) of EPFRs by age groups *via* inhalation of $PM_{2.5}$.

Age	HA		TR		AR	
	max	median	max	median	max	median
0−3 m	1.11	0.21	0.12	0.02	0.25	0.05
3−6 m	2.06	0.40	0.22	0.04	0.46	0.09
6−9 m	2.70	0.52	0.29	0.06	0.60	0.12
9 m−1 y	3.14	0.60	0.33	0.06	0.70	0.13
1−2 y	3.02	0.58	0.32	0.06	0.67	0.13
2−3 y	2.79	0.54	0.30	0.06	0.62	0.12
3−4 y	2.96	0.57	0.31	0.06	0.66	0.13
4−5 y	2.53	0.49	0.27	0.05	0.56	0.11
5−6 y	2.32	0.45	0.25	0.05	0.51	0.10
6−9 y	1.55	0.30	0.16	0.03	0.34	0.07
9−12 y	1.48	0.28	0.16	0.03	0.33	0.06
12−15 y	1.14	0.22	0.12	0.02	0.25	0.05
15−18 y	0.97	0.19	0.10	0.02	0.21	0.04
18−44 y	2.53	0.49	0.27	0.05	0.56	0.11
45−59 y	2.69	0.52	0.28	0.05	0.60	0.11
60−79 y	2.14	0.41	0.23	0.04	0.47	0.09
80 y+	1.52	0.29	0.16	0.03	0.34	0.06

Abbreviations: HA, head airway; TR, tracheobronchial region; AR, alveolar region; DED, the daily exposure dose.

Table S22. EQ of EPFRs by age groups *via* inhalation of $PM_{2.5}$.

Age	HA		TR		AR	
	max	median	max	median	max	median
0−3 m	0.33	0.06	0.03	0.01	0.07	0.01
3−6 m	0.42	0.08	0.04	0.01	0.09	0.02
6−9 m	0.48	0.09	0.05	0.01	0.11	0.02
9 m−1 y	0.52	0.10	0.06	0.01	0.12	0.02
1−2 y	0.51	0.10	0.05	0.01	0.11	0.02
2−3 y	0.56	0.11	0.06	0.01	0.12	0.02
3−4 y	0.71	0.14	0.08	0.01	0.16	0.03
4−5 y	0.75	0.14	0.08	0.02	0.17	0.03
5−6 y	0.78	0.15	0.08	0.02	0.17	0.03
6−9 y	0.90	0.17	0.10	0.02	0.20	0.04
9−12 y	1.17	0.23	0.12	0.02	0.26	0.05
12−15 y	1.20	0.23	0.13	0.02	0.27	0.05
15−18 y	1.24	0.24	0.13	0.03	0.28	0.05
18−44 y	1.48	0.29	0.16	0.03	0.33	0.06
45−59 y	1.48	0.29	0.16	0.03	0.33	0.06
60−79 y	1.23	0.24	0.13	0.02	0.27	0.05
80 y+	1.07	0.21	0.11	0.02	0.24	0.05

Abbreviations: EQ, the number of equivalent cigarettes.

References

(1) Tahir, A.; Abbasi, N.A.; He, C.; Ahmad, S.R.; Baqar, M.; Qadir, A. Spatial distribution and ecological risk assessment of short and medium chain chlorinated paraffins in water and sediments of river Ravi, Pakistan. *Sci Total Environ* **2024**, 926171964.

(2) Hinds, W.C. Aerosol Technology Properties, Behavior, and Measurement of Airborne Particles second ed. John Wiley & Sons, Inc. New York., **1999**.

(3) Bian, R.; Stubbings, W.A.; Li, F.; Wu, F.; Wang, S. Distribution, Partitioning, Source Apportionment, and Ecological Risk Assessment of Legacy and Emerging PFAS in Water and Sediment of the Pearl River Delta. *Environ Sci Technol Water* **2025**, 5(1), 253-263.

(4) Zhang, X.; Zhang, Z.F.; Zhang, X.; Zhu, F.J.; Li, Y.F.; Cai, M.; Kallenborn, R. Polycyclic Aromatic Hydrocarbons in the Marine Atmosphere from the Western Pacific to the Southern Ocean: Spatial Variability, Gas/Particle Partitioning, and Source Apportionment. *Environ Sci Technol* **2022**, 56(10), 6253-6261.

(5) Ikemori, F.; Uranishi, K.; Asakawa, D.; Nakatsubo, R.; Makino, M.; Kido, M.; Mitamura, N.; Asano, K.; Nonaka, S.; Nishimura, R.; Sugata, S. Source apportionment in $PM_{2.5}$ in central Japan using positive matrix factorization focusing on small-scale local biomass burning. *Atmos Pollut Res* **2021**, 12(3), 162-172.

(6) Norris, G., Duvall, R., Brown, S., & Bai, S. EPA positive matrix factorization (PMF) 5.0 fundamentals and user guide, US Environmental Protection Agency, Washington, DC. www2. epa. gov/sites/production/files/2015-02/documents/pmf_5. 0_user_guide. pdf, **2014**.

(7) Gehling, W.; Khachatryan, L.; Dellinger, B. Hydroxyl Radical Generation from Environmentally Persistent Free Radicals (EPFRs) in PM2.5. *Environmental Science & Technology* **2014**, 48(8), 4266-4272.

(8) Shah, S.; Chen, J.; Saleem, A.R.; Sun, Y.; Ai, J.; Huang, H.; Zhang, L.; Khan, C. Source-oriented pollution characteristics and decay kinetics of environmentally persistent free radicals in $PM_{2.5}$ and PM_{10}. *Journal of Hazardous Materials* **2025**, 495139074.

(9) Zhao, X., & Duan, X. Highlights of the Chinese Exposure Factors Handbook (Adults), first ed. China Environmental Science Press. Beijing, **2016**.

(10) Zhao, X., & Duan, X. Report of Environmental Exposure Related Activity Patterns Research of Chinese Population (Children), first ed. China Environmental Science Press. Beijing, **2016**.

(11) Chaemfa, C.; Xu, Y.; Li, J.; Chakraborty, P.; Hussain Syed, J.; Naseem Malik, R.; Wang, Y.; Tian, C.; Zhang, G.; Jones, K.C. Screening of atmospheric short- and medium-chain chlorinated paraffins in India and Pakistan using polyurethane foam based passive air sampler. *Environ Sci Technol* **2014**, 48(9), 4799-4808.

(12) Tahir, A.; Abbasi, N.A.; He, C.; Ahmad, S.R. Spatial distribution and air-soil exchange of short and medium chain chlorinated paraffins in Lahore, Pakistan. *Sci Total Environ* **2024**, 953176054.

(13) Diefenbacher, P.S.; Bogdal, C.; Gerecke, A.C.; Glüge, J.; Schmid, P.; Scheringer, M.; Hungerbühler, K. Short-Chain Chlorinated Paraffins in Zurich, Switzerland—Atmospheric Concentrations and Emissions. *Environ Sci Technol* **2015**, 49(16), 9778-9786.

(14) He, C.; Thai, P.K.; Bertrand, L.; Jayarathne, A.; van Mourik, L.; Phuc, D.H.; Banks, A.; Mueller, J.F.; Wang, X.F. Calibration and Application of PUF Disk Passive Air Samplers To Assess Chlorinated Paraffins in Ambient Air in Australia, China, and Vietnam. *Environ Sci Technol* **2023**, 57(50), 21061-21070.

(15) Jiang, L.; Gao, W.; Ma, X.; Wang, Y.; Wang, C.; Li, Y.; Yang, R.; Fu, J.; Shi, J.; Zhang, Q.; Wang, Y.; Jiang, G. Long-Term Investigation of the Temporal Trends and Gas/Particle Partitioning of Short- and Medium-Chain Chlorinated Paraffins in Ambient Air of King George Island, Antarctica. *Environ Sci Technol* **2021**, 55(1), 230-239.

(16) Niu, S.; Harner, T.; Chen, R.; Parnis, J.M.; Saini, A.; Hageman, K. Guidance on the Application of Polyurethane Foam Disk Passive Air Samplers for Measuring Nonane and Short-Chain Chlorinated Paraffins in Air: Results from a Screening Study in Urban Air. *Environ Sci Technol* **2021**, 55(17), 11693-11702.

(17) Balla, D.; Costopoulou, D.; Perkons, I.; Saraga, D.; Zacs, D.; Voutsa, D.; Leondiadis, L.; Maggos, T. Short- and medium-chain polychlorinated alkanes in the air of Athens, Greece. *Chemosphere* **2025**, 373144162.

(18) Al Saify, I.; Brandsma, S.H.; van Mourik, L.M.; Eckhardt, S.; Bohlin-Nizzetto, P.; Warner, N.A. Physical and chemical processes driving remote seasonal atmospheric exposure to cyclic volatile methysiloxanes and short-chain chlorinated paraffins. *Atmos Environ* **2023**, 304, Article 119754.

(19) Wang, T.; Han, S.; Yuan, B.; Zeng, L.; Li, Y.; Wang, Y.; Jiang, G. Summer-winter concentrations and gas-particle partitioning of short chain chlorinated paraffins in the atmosphere of an urban setting. *Environ Pollut* **2012**, 17138-45.

(20) Li, T.; Gao, S.; Ben, Y.; Zhang, H.; Kang, Q.; Wan, Y. Screening of Chlorinated Paraffins and Unsaturated Analogues in Commercial Mixtures: Confirmation of Their Occurrences in the Atmosphere. *Environ Sci Technol* **2018**, 52(4), 1862-1870.

(21) Liu, D.; Li, Q.; Cheng, Z.; Li, K.; Li, J.; Zhang, G. Spatiotemporal variations of chlorinated paraffins in $PM_{2.5}$ from Chinese cities: Implication of the shifting and upgrading of its industries. *Environ Pollut* **2020**, 259113853.

(22) Li, H.; Li, J.; Li, H.; Yu, H.; Yang, L.; Chen, X.; Cai, Z. Seasonal variations and inhalation risk assessment of short-chain chlorinated paraffins in $PM_{2.5}$ of Jinan, China. *Environ Pollut* **2019**, 245325-330.

(23) Huang, J.; Zhao, L.; Shi, Y.; Zeng, X.; Sun, W.; Zhao, X.; Liu, R.; Wu, Q.; Dong, G.; Chen, D.; Liu, X. Characterization of short-, medium- and long-chain chlorinated paraffins in ambient $PM_{2.5}$ from the Pearl River Delta, China. *Environ Int* **2023**, 175107932.

(24) Zhou, T.; Yang, Q.; Weng, J.; Gao, L.; Liu, Y.; Xu, M.; Zhao, B.; Zheng, M. Characterization and health risks of short- and medium-chain chlorinated paraffins in the gas and size-fractionated particulate phases in ambient air. *Chemosphere* **2024**, 358142225.

(25) He, Q.Y.; Guo, Y.; Mao, H.Y.; Chen, H.; Li, Y.; Zhang, W.F.; Zhang, Y.H.; Zhao, W.D. Ultrasonic-assisted Solvent Extraction Method for the Determination of Environmentally Persistent Free Radicals in $PM_{2.5}$. *Chemistry Letters* **2021**, 50(7), 1368-1371.

(26) Zhang, X.; Wang, Y.; Yao, K.; Zheng, H.; Guo, H. Oxidative potential, environmentally persistent free radicals and reactive oxygen species of size-resolved ambient particles near highways. *Environ Pollut* **2024**, 341122858.

(27) Li, Z.; Zhao, H.; Li, X.; Bekele, T.G. Characteristics and sources of environmentally persistent free radicals in $PM_{2.5}$ in Dalian, Northeast China: correlation with polycyclic aromatic hydrocarbons. *Environ Sci Pollut Res Int* **2022**, 29(17), 24612-24622.

(28) Ainur, D.; Chen, Q.; Wang, Y.; Li, H.; Lin, H.; Ma, X.; Xu, X. Pollution characteristics and sources of environmentally persistent free radicals and oxidation potential in fine particulate matter related to city lockdown (CLD) in Xi'an, China. *Environ Res* **2022**, 210112899.

(29) Ai, J.; Qin, W.; Chen, J.; Sun, Y.; Yu, Q.; Xin, K.; Huang, H.; Zhang, L.; Ahmad, M.; Liu, X. Pollution characteristics and light-driven evolution of environmentally persistent free radicals in $PM_{2.5}$ in two typical northern cities of China. *J Hazard Mater* **2023**, 454131466.

(30) Feng, W.; Zhang, Y.; Huang, L.; Li, Y.; Guo, Q.; Peng, H.; Shi, L. Spatial distribution, pollution characterization, and risk assessment of environmentally persistent free radicals in urban road dust from central China. *Environ Pollut* **2022**, 298118861.

(31) Xiao, K.; Wang, Z.; Zhou, Y.; Fu, D.; Zhang, Y.; Luo, Z.; Lin, Y.; Wang, Q.; Pei, J.; Shen, G. Size-resolved environmentally persistent free radicals in urban road dust and association with transition metals. *Environ Geochem Health* **2023**, 45(11), 7829-7839.

(32) Xiao, K.; Lin, Y.; Wang, Q.; Lu, S.; Wang, W.; Chowdhury, T.; Enyoh, C.E.; Rabin, M.H. Characteristics and Potential Inhalation Exposure Risks of Environmentally Persistent Free Radicals in Atmospheric Particulate Matter and Solid Fuel Combustion Particles in High Lung Cancer Incidence Area, China. *Atmosphere* **2021**, 12(11), 1467.

(33) Yang, X.; Liu, F.; Yang, S.; Yang, Y.; Wang, Y.; Li, J.; Zhao, M.; Wang, Z.; Wang, K.; He, C.; Tong, H. Atmospheric evolution of environmentally persistent free radicals in the rural North China Plain: effects on water solubility and $PM_{2.5}$ oxidative potential. *Atmos Chem Phys* **2024**, 24(19), 11029-11043.

(34) Wu, S.; Xin, K.; Chen, J.; Dambajamts, N.; Sun, Y.; Ai, J.; Ouyang, W.; Ulziibat, B.; Batkhuyag, E.U.; Tseren-Ochir, S.E. Seasonal variations and intercorrelations of polycyclic aromatic hydrocarbons, heavy metals and environmentally persistent free radicals in $PM_{2.5}$ in Ulaanbaatar, Mongolia. *J Hazard Mater* **2025**, 489137586.

(35) Guo, C.; Hasan, F.; Lay, D.; Dela Cruz, A.L.N.; Ghimire, A.; Lomnicki, S.M. Phytosampling-a supplementary tool for particulate matter (PM) speciation characterization. *Environ Sci Pollut Res Int* **2021**, 28(29), 39310-39321.

(36) Ahmad, M.; Chen, J.; Yu, Q.; Tariq Khan, M.; Weqas Ali, S.; Nawab, A.; Phairuang, W.; Panyametheekul, S. Characteristics and Risk Assessment of Environmentally Persistent Free Radicals (EPFRs) of $PM_{2.5}$ in Lahore, Pakistan. *Int J Environ Res Public Health* **2023**, 20(3).
